# Reclassifying BRCA1 c.4358-2A > G and BRCA2 c.475 + 5G > C variants from “Uncertain Significance” to “Pathogenic” based on minigene assays and clinical evidence

**DOI:** 10.1007/s00432-023-05597-y

**Published:** 2024-02-01

**Authors:** Ying Ning, Yu Zhang, Tian Tian, Yu Chen, Jia Wang, Ke Lei, Zhumei Cui

**Affiliations:** 1 Department of Clinical Medicine, Qingdao University, Qingdao, 266003 China; 2 Department of Obstetrics and Gynecology, The Affiliated Hospital of Qingdao University, No. 59 Haier Road, Laoshan District, Qingdao, 266000 China; 3 Center of Tumor Immunology and Cytotherapy, Medical Research Center, The Affiliated Hospital of Qingdao University, No. 59 Haier Road, Laoshan District, Qingdao, 266000 China

**Keywords:** Ovarian neoplasm, BRCA1/2, Intronic variant, Splice

## Abstract

**Background:**

Pathogenic variants in BRCA genes play a crucial role in the pathogenesis of ovarian cancer. Intronic variants of uncertain significance (VUS) may contribute to pathogenicity by affecting splicing. Currently, the significance of many intronic variants in BRCA has not been clarified, impacting patient treatment strategies and the management of familial cases.

**Method:**

A retrospective study was conducted to analyze BRCA intronic VUS in a cohort of 707 unrelated ovarian cancer patients at a single institution from 2018 to 2023. Three splicing predictors were employed to analyze detected intronic VUS. Variants predicted to have splicing alterations were selected for further validation through minigene assays. Patient and familial investigations were conducted to comprehend cancer incidence within pedigrees and the application of poly (ADP-ribose) polymerase inhibitors (PARPi) by the patients. In accordance with the guidelines of the American College of Medical Genetics and Genomics (ACMG), the intronic VUS were reclassified based on minigene assay results and clinical evidence.

**Result:**

Approximately 9.8% (69/707) of patients were identified as carriers of 67 different VUS in BRCA1/2, with four intronic variants accounting for 6% (4/67) of all VUS. Splicing predictors indicated potential splicing alterations in splicing for BRCA1 c.4358-2A>G and BRCA2 c.475+5G>C variants. Minigene assays utilizing the pSPL3 exon trapping vector revealed that these variants induced changes in splicing sites and frameshift, resulting in premature termination of translation (p. Ala1453Glyfs and p. Pro143Glyfs). According to ACMG guidelines, BRCA1 c.4358-2A>G and BRCA2 c.475+5G>C were reclassified as pathogenic variants. Pedigree investigations were conducted on patients with BRCA1 c.4358-2A>G variant, and the detailed utilization of PARPi provided valuable insights into research on PARPi resistance.

**Conclusion:**

Two intronic VUS were reclassified as pathogenic variants. A precise classification of variants is crucial for the effective treatment and management of both patients and healthy carriers.

**Supplementary Information:**

The online version contains supplementary material available at 10.1007/s00432-023-05597-y.

## Introduction

Germline variants of BRCA1/2 have been demonstrated as significant pathogenic factors in hereditary breast and ovarian cancer syndrome (HBOC) (Ponti et al. 2023). As the number of genetic tests continues to rise, an increasing number of pathogenic variants are being identified, which contribute to a lifetime risk of breast cancer (BC), ovarian cancer (OC), and other cancers (Jimenez-Sainz et al. 2021). The American College of Medical Genetics and Genomics (ACMG) guidelines provide a classification system for BRCA gene sequence variants, dividing them into five categories: "pathogenic," "likely pathogenic," "VUS (variants of uncertain significance)," "likely benign," and "benign" (Richards et al. 2015). Definitive examination results hold significant implications for patient treatment, prognosis, as well as prevention and management for family members (Kuchenbaecker et al. 2017; Easton et al. 1997). However, up to 20% of BRCA1/2 tests will report genetic VUS (Eccles et al. 2015), thus impeding the clinical decision-making.

VUS refers to a change in the nucleotide sequence of DNA that produces inconclusive results regarding the loss of normal function of the corresponding protein or the potential risk of developing a disease (Fanale et al. 2022). Among VUS, intron variants occupy a certain proportion, they can potentially disrupt the regular splicing process of genes during transcription, leading to alterations in the resultant protein structure. In terms of splicing, intronic variants, particularly those occurring at or near the classical splice sites, are the most common type of splice-affecting variants. In the past, more attention was paid to variants in the coding region, but with the advancement of sequencing technology and functional analysis methods, an increasing number of pathogenic intron variants that cause splicing changes have been identified (Hoberg-Vetti et al. 2020; Reuter et al. 2023; Sanz et al. 2010; Fraile-Bethencourt et al. 2019; Valenzuela-Palomo et al. 2022). Splicing variants can have significant effects on protein sequence, structure, and function, and are frequently observed in various genetic diseases (Reuter et al. 2023; Wang and Cooper 2007). It is crucial to identify pathogenic splice variants for supporting their clinical interpretation. The ACMG guidelines consider functional studies as strong evidence to determine the pathogenicity of a specific variant, using the codes "PS3"/"BS3" (accepted functional analysis demonstrates [destructive/non-destructive] effects on a gene or gene product) (Richards et al. 2015).

In this study, we presented a report on BRCA intronic variants in 707 unrelated cases of OC patients and subjected these variants to three in silico splice predictors. Minigene assays were conducted to validate the splicing and translation changes of variants predicted to have splicing alteration. We reclassified the variants and discussed the utilization of poly (ADP-ribose) polymerase inhibitors (PARPi) in the OC patients with the variant, aiming to offer precise guidance for clinical decision-making.

## Methods

### Patients and clinical information

We retrospectively reviewed BRCA1/2 testing results of 707 unrelated patients with primary OC in Affiliated Hospital of Qingdao University from June 1, 2018, to March 1, 2023. The family histories of these patients were followed up via telephone. Ethical approval has been obtained from the Ethics Committee of Affiliated Hospital of Qingdao University.

### BRCA1/2 testing, nomenclature, and classification

Briefly, DNA was extracted from 2 ml of peripheral blood from each patient. Next-generation sequencing technology on the Illumina MiniSeq was utilized to detect variants of BRCA1 and BRCA2 across all coding exons and exon–intron boundaries. The nomenclature of variants followed the guidelines of the Human Genome Variation Society (HGVS), with the variants referenced as NM_007294.4 for BRCA1 and NM_000059.4 for BRCA2. The identified BRCA1/2 variations were classified according to the 2015 ACMG guidelines (Richards et al. 2015). To determine whether the detected variants had been previously reported, we searched the ClinVar database (http://www.ncbi.nlm.nih.gov/clinvar/) (Li and Wang 2017) and BRCA Exchange database (http://brcaexchange.org). We used the Genome Aggregation Database (GnomAD, https://gnomad.broadinstitute.org/) to estimate the frequency of variants in diverse populations.

### Functional prediction of VUS

The prediction of splicing alterations was performed with SpliceAI (Jaganathan et al. 2019) lookup tool (The δ score ranges from 0.11 to 0.99, https://spliceailookup.broadinstitute.org/, accessed on 25 March 2023), MaxEntScan (Yeo and Burge 2004) (for 3′ splice sites: http://hollywood.mit.edu/burgelab/maxent/Xmaxentscan_scoreseq_acc.html and for 5′ splice sites: http://hollywood.mit.edu/burgelab/maxent/Xmaxentscan_scoreseq.html) using the maximum entropy model, and NNSplice (Reese et al. 1997) (https://fruitfly.org/seq_tools/splice.html).

### Minigene assays

#### Wild-type and mutated minigene construct

To construct the minigene assays, we followed established procedures utilizing the pSPL3 exon-trapping vector, as outlined in a previous study (Sanz et al. 2010; Weisschuh et al. 2012). For the BRCA1 c.4358-2A > G variant, we initially amplified BRCA1 exon 13 along with its 200 bp upstream and downstream intronic regions from a healthy human genome, employing specific primers. The ClonExpress II One Step Cloning Kit (C112-01, Vazyme, Nanjing, China) was employed to insert these amplified fragments into the pSPL3 vector, thereby creating the wild-type minigene. For the c.4358-2A > G point mutant, a linear plasmid was generated through amplification using site-specific mutant primers, based on the wild-type minigene. For the BRCA2 c.475 + 5G > C variant, a wild-type minigene was established using BRCA2 exon 5, along with its 200 bp upstream and 89 bp downstream intronic regions (the downstream of exon 5 only contains intronic regions of 89 bp). The minigene for BRCA2 c.475 + 5G > C was then constructed through site-specific mutation. Subsequently, minigene-containing plasmids were transferred to DH5α competent cells and cultivated overnight at 37 °C on LB solid medium supplemented with ampicillin. The following day, single colonies were sequenced by the Beijing Genomics Institute.

#### Cell culture and transfection

HEK293T cells were cultured in their dedicated medium (Wuhan Procell Life Science and Technology Co. Ltd., Wuhan, China) at 37 °C in an incubator with 5% CO_2_. After 48 h, HEK293T cells were seeded and transfected with the respective pSPL3_wild-type and mutation vectors using lipofectamine 8000 (C0533, Beyotime Biotechnology, Shanghai, China). The empty vector pSPL3 served as the negative control.

#### Product analysis

Total RNA was extracted and reverse transcribed to cDNA using the Hifair^®^ III 1st-Strand cDNA Synthesis SuperMix (11141ES10, YEASEN, Shanghai, China). Subsequently, pSPL3 vector-specific primers were employed for PCR amplification. The resulting PCR products were separated by electrophoresis on a 1.8% agarose gel and subsequently sequenced to analyze the nucleic acid changes. All the primers used in the minigene assays are provided in supplementary material 1.

### Homology modeling of protein structure

The BRCA1 structure (UniProt: P38398) was obtained from the AlphaFold Protein Structure Database (Jumper et al. 2021; Varadi et al. 2022). Homology modeling of BRCA1_G1453A was performed using the SWISS-MODEL server (Waterhouse et al. 2018; Bienert et al. 2017). By inputting the FASTA sequence of BRCA1_G1453A, a monomeric structure of the BRCA1_G1453A protein was generated with a Global Model Quality Estimate (GMQE) of 0.36.

## Result

### VUS of BRCA1/2 in 707 OC patients

We conducted a retrospective review of BRCA1/2 testing results for 707 patients diagnosed with primary OC. Among these patients, a total of 69 (9.8%) were found to carry 67 different VUS in BRCA1/2 (Supplementary material 1). Within these 67 VUS of BRCA1/2, we identified 4 intronic variants (BRCA1: c.4358-2A > G; BRCA2: c.475 + 5G > C, c.1909 + 22delT, c.7618-15_7618-14del), which accounted for 6% (4/67) of all VUS. Two of them (BRCA2: c.475 + 5G > C, c.7618-15_7618-14del) were novel variants that had never been reported before.

We utilized three in silico prediction tools (SpliceAI, MaxEntScan, NNSplice) to assess the splicing effects of the four VUS (Table 1). Notably, one intronic variant, BRCA1 c.4358-2A > G, consistently exhibited splice-affecting results across all three predictors. Additionally, another variant, BRCA2 c.475 + 5G > C, appeared to have the potential for splicing alterations. Consequently, we conducted in vitro minigene assays to validate these predictions.

**Table 1:** Intronic mutant spectrum of VUS

Gene name	cDNA change	Freq	Intron	BRCA Exchange	ClinVar	GnomAD	SpliceAI (Δ score)/pre-mRNA position	NNSplice % decrease of splice site strength	MaxEntScan % decrease of splice site strength
BRCA1	c.4358-2A > G	2	Intron13	NR	VUS	None	Ass loss (0.8)/ – 2 bp ASS gain (0.97)/ – 1 bp	– 100%	– 100%
BRCA2	c.475 + 5G > C	1	Intron5	Novel	Novel	None	DSS loss (0.24)/ – 5 bp	– 14%	– 26%
BRCA2	c.1909 + 22delT	1	Intron10	NR	VUS	None	No effect	No effect	No effect
BRCA2	c.7618-15_7618-14del	1	Intron15	Novel	Novel	None	No effect	+ 3%	No effect

*Freq* Frequence, *NR* Not Yet Reviewed, *VUS* Variants of Uncertain Significance

### Minigene assays to validate the splicing alteration

**Fig. 1:**
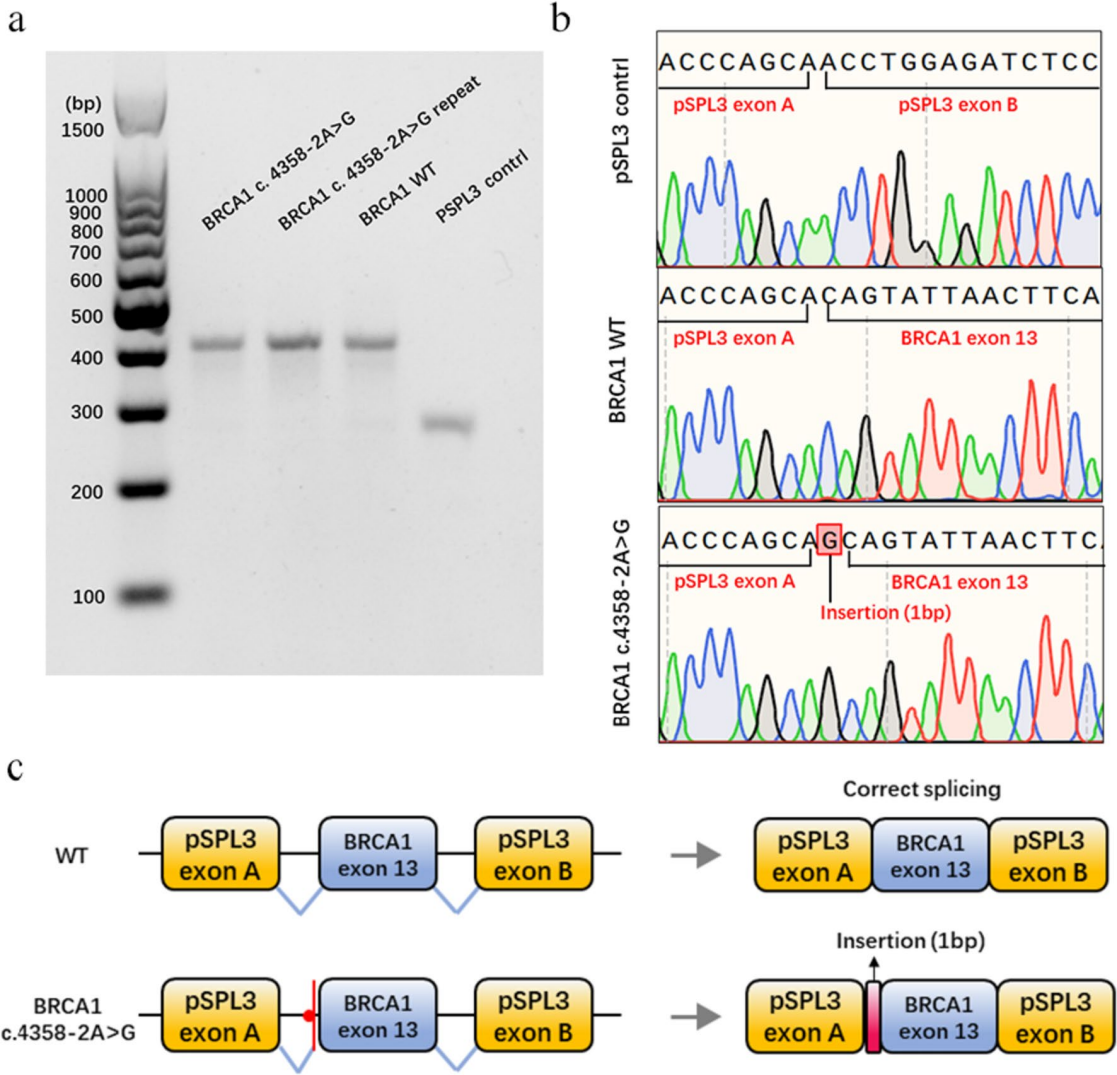
Splicing change of BRCA1 c.4358-2A > G. **a** Nucleic acid electrophoresis of the RT-PCR products. **b** Sequencing analysis of the RT-PCR products. **c** Schematic diagram of the splicing change of BRCA1 c.4358-2A > G

**Fig. 2:**
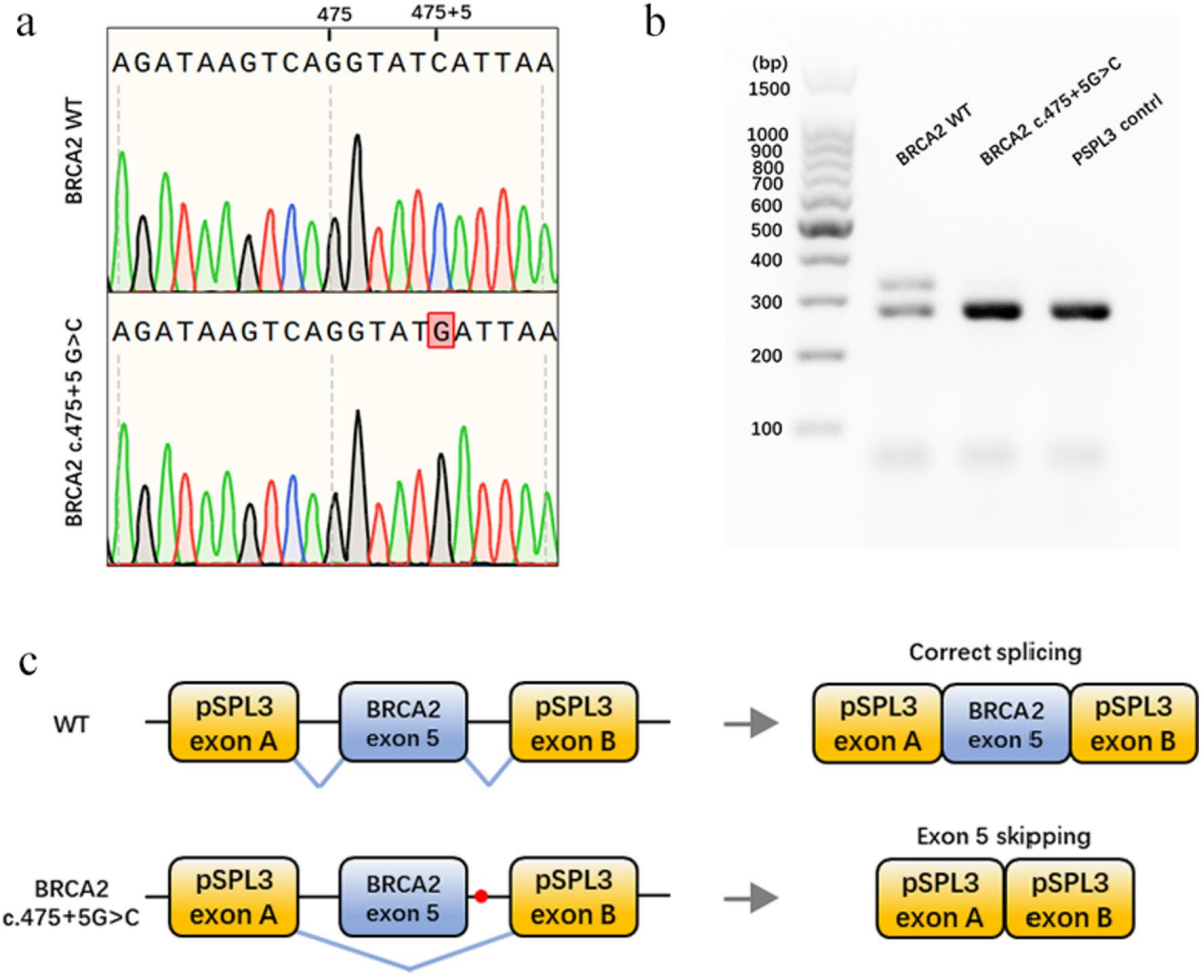
Splicing change of BRCA2 c.475 + 5G > C. **a** Sequencing results of wild-type and mutant minigenes. **b** Agarose gels of the RT-PCR products. **c** Schematic diagram of the splicing change of BRCA2 c.475 + 5G > C

The pSPL3 splicing reporter minigene assays were subsequently conducted to assess the splicing alterations of BRCA1 c.4358-2A > G and BRCA2 c.475 + 5G > C. For the variant of BRCA1 c.4358-2A > G, as demonstrated in Fig. 1a, both the wild-type and the mutant plasmids produced single RT-PCR products, indicating no change in length. However, the RT-PCR products were subsequently sequenced to analyze the nucleic acid changes, and a 1-bp splicing frameshift was detected in the mutant variant (r.4357_4358insG) (Fig. 1b, c). In the case of the BRCA2 c.475 + 5G > C variant, we observed transcripts that skipped exon 5 of BRCA2 (r.426_475del), resulting in an in-frame deletion (Fig. 2a–c).

### Transcript analysis and protein structure homology modeling

**Fig. 3:**
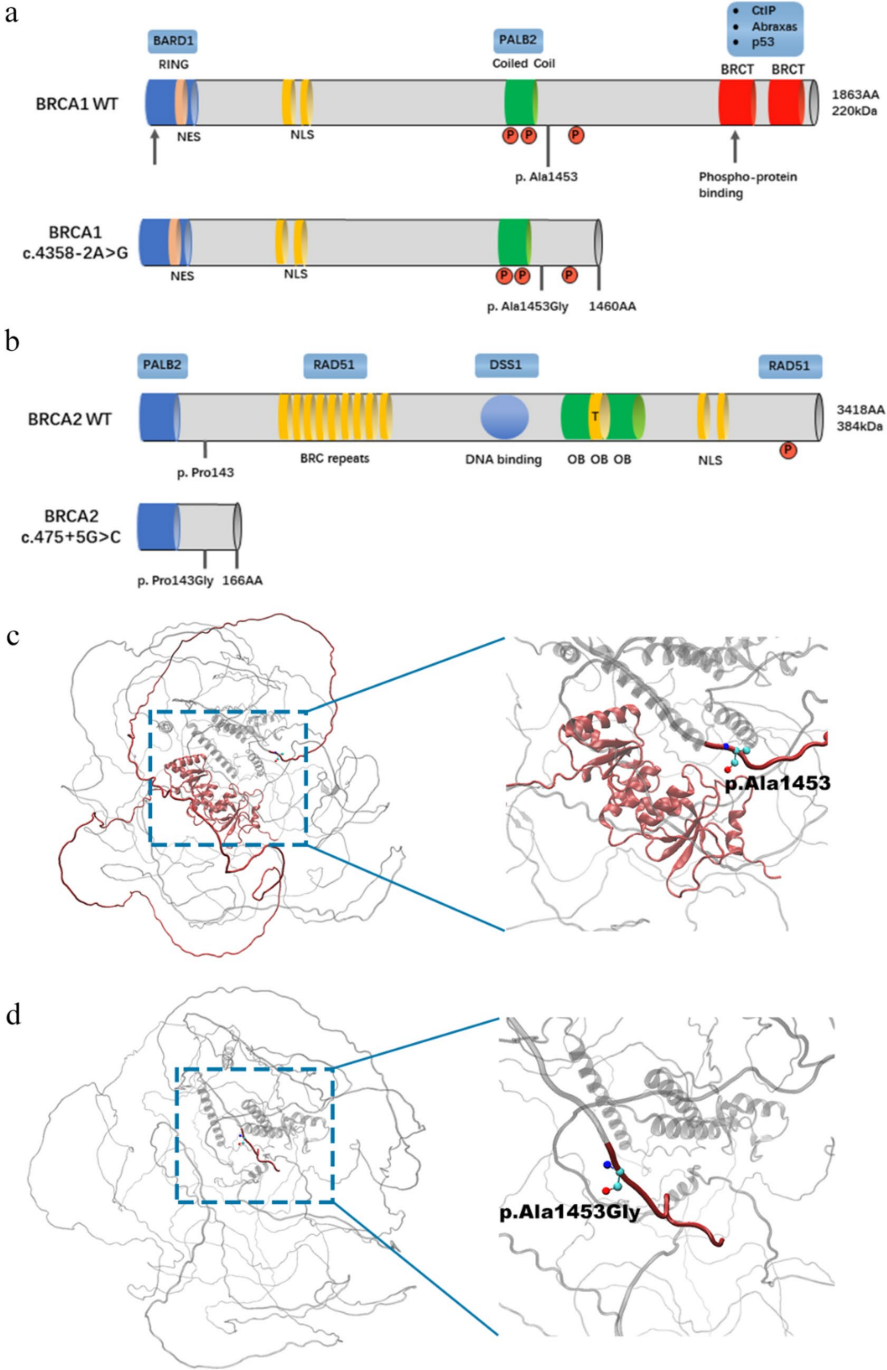
Prediction of protein structural changes. **a** Schematic diagram of truncated mutant of BRCA1 c.4358-2A > G; **b** Schematic diagram of truncated mutant of BRCA2 c.475 + 5G > C; Homology modeling of protein structures of BRCA1 (UniProt: P38398) (**c**) and BRCA1_A1453G (**d**), with the amino acid residues at the A1453-C-terminus and G1453-C-terminus labeled in red, where different amino acids in site of 1453 are labeled with the ball-and-stick model

We analyzed the translation sequence after frameshift splicing and found that both variants caused early termination of translation after frameshift (p. Ala1453Glyfs, p. Pro143Glyfs) (Fig. 3a, b, Supplementary material 2). We referred to the BRCA1 protein model in the AlphaFold Protein Structure Database and employed the SWISS-MODEL server for homology modeling of the mutated protein. The predicting structure of wild-type BRCA1 protein is exhibited in Fig. 3c, with the red region representing the protein structure after amino acid 1453. However, in the c.4358-2A > G mutated protein model (Fig. 3d), amino acid 1453 is altered to glycine, resulting in the loss of important functional domains (indicated by the blue box) and changes in the protein's spatial structure. For BRCA2 c.475 + 5G > C variant, no additional modeling was conducted, as translation terminates at an early stage.

### Characteristics and pedigrees of patients

**Fig. 4:**
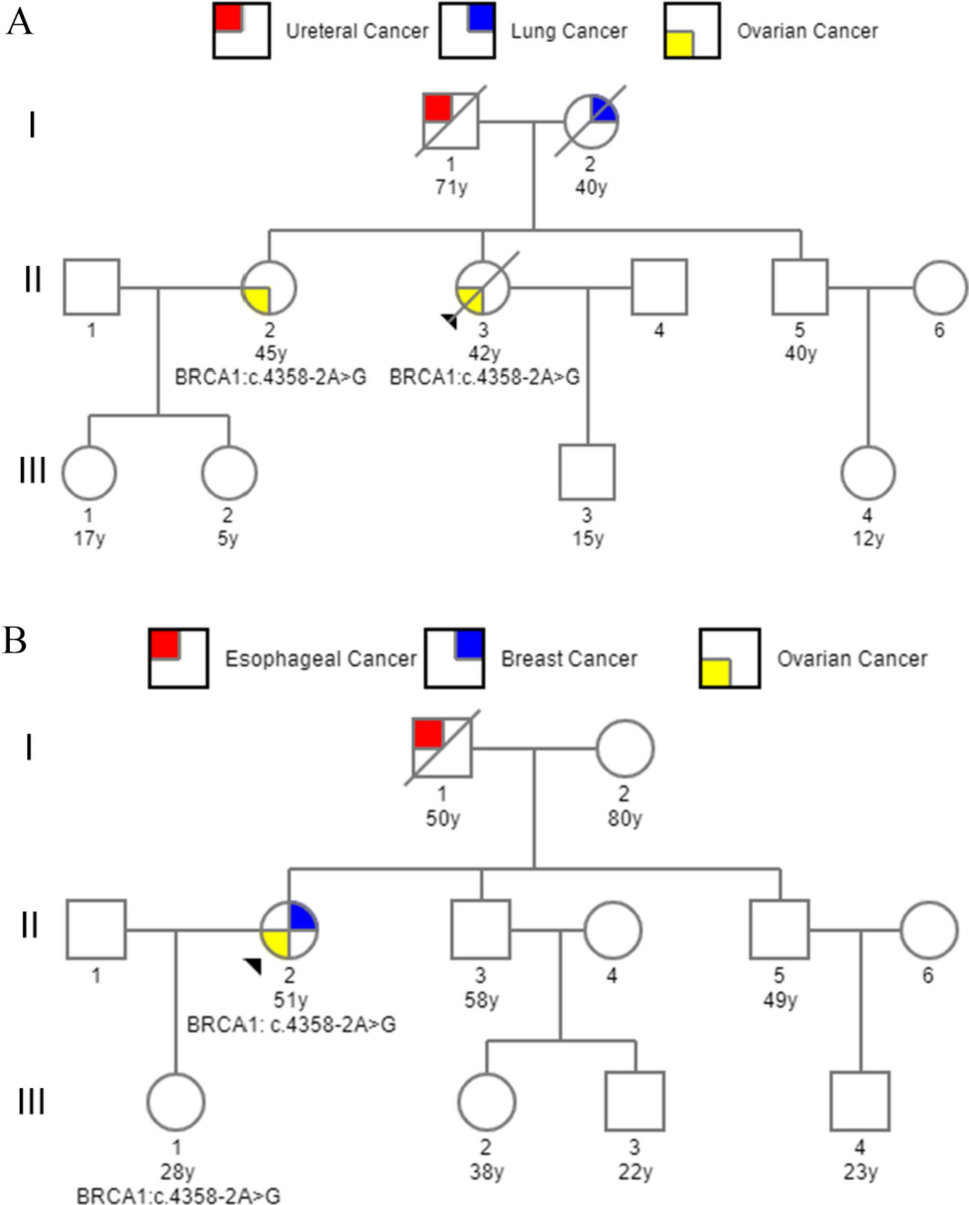
Pedigree of the two families (**A** and **B**) with variant of BRCA1 c.4358-2A > G

We analyzed the pedigrees of two OC patients harboring BRCA1 c.4358-2A > G variant (A II:2 and B II:2 in Fig. 4), and identified another OC patient (A II:3) with the same variant by pedigree investigation. Patient A II:2 and A II:3 suffered a single type of cancer, while patient B II:2 underwent two cancer types, BC and OC. She received surgery, chemotherapy, and endocrine therapy for cure of BC at the age of 33, which was 13 years before the onset of OC (Table 2). The pedigrees revealed that the parents of the two probands had experienced distinct types of cancer, including esophageal, lung, and ureteral cancers. To investigate the applicability of PARPi in patients with this variant, we compiled the clinical data of the three OC patients from the aforementioned pedigrees. In family A, patients A II:2 and A II:3 received olaparib following recurrent chemotherapy but experienced recurrence after 8 and 6 months, respectively. Unfortunately, despite ongoing treatment for two years following the second recurrence, patient A II:3 ultimately passed away in September 2022 due to multiple organ failure. On the other hand, proband B II:2 underwent maintenance therapy with olaparib after first-line chemotherapy, which yielded favorable outcomes. The patient discontinued olaparib after 3 years and remained relapse-free until the end of the observation period.

**Table 2:** Clinicopathological characteristics of OC patients with BRCA1 c.4358-2A > G variant

Patient	Age of onset	Stage	Pathology	First-line chemotherapy	PFS (m)	Occasion of PARPi application	Time interval for recurrence after PARPi application (m)
A II:2	41	IIIC	Serous adenocarcinoma	PC*6	8	Maintenance therapy after chemotherapy for recurrence	8
A II:3	37	IIIC	Serous adenocarcinoma	PC*6	12	Maintenance therapy after chemotherapy for recurrence	6
B II:2	46 (OC)	IIIC	Serous adenocarcinoma	PC*6	55	Maintenance therapy after first-line chemotherapy	–
	33 (BC)	I	Ductal carcinoma	PC*6	211	Tamoxifen (3 years for maintenance therapy after first-line chemotherapy)	

*PC* Paclitaxel + Carboplatin

## Discussion

HBOC-associated BRCA1 and BRCA2 genes have been discovered for more than two decades (Miki et al. 1994; Wooster et al. 1995). Patients with pathogenic or likely pathogenic variants show better response to PARPi, and carriers of pathogenic or likely pathogenic variants can choose to undergo prophylactic surgery or intensive screening (Finch et al. 2014). However, the clinical relevance of VUS remains uncertain and cannot serve as a basis for guiding patient and familial management (Domchek et al. 2013).

While the majority of VUS are typically found in the coding region of exons, it is important to note that some of the variants located in introns with uncertain significance can also be reclassified as pathogenic or likely pathogenic variants after validation (Reuter et al. 2023). The BRCA exchange database has identified numerous intronic BRCA variants as pathogenic, yet there are still over 40,000 intronic variants that remain uncharacterized. In 707 unrelated OC patients of our study, four intronic variants were initially classified as VUS. Despite the splicing predictors consistently indicated that BRCA1 c.4358-2A > G and BRCA2 c.475 + 5G > C resulted in abnormal splicing, it is undeniable that the classification of VUS did not provide meaningful guidance for patients' treatment decisions and family management. In order to clarify its clinical significance, functional analysis was performed for this variant, which are considered by the ACMG/AMP guidelines as the strong evidence to evaluate the pathogenicity of the variant. As expected, the variant of the BRCA1 c.4358-2A > G induced a change in the splicing site and caused frameshift in the translation region. We attempted to amplify cDNA fragments of BRCA1 from patient B II:2, but no product was obtained, which may be attributed to nonsense-mediated decay (NMD) (Perrin-Vidoz et al. 2002). According to the guidelines of ACMG, BRCA1 c.4358-2A > G variant can be reclassified as a pathogenic variant (PVS1 + PS3_supporting + PM2 + PP3 + PP4) (Abou Tayoun et al. 2018). Our findings indicate that this variant has been previously reported in the Chinese population, suggesting a potentially higher prevalence among individuals of Chinese descent (Table 3) (Moradian et al. 2021; Hu et al. 2022). For BRCA2 c.475 + 5G > C, the minigene assay revealed the skipping of exon 5, inducing a frameshift in the translational region. In our experiments, the wild-type minigene was constructed solely with exon 5 and its upstream and downstream introns. The absence of natural flanking exons might result in partial exon 5 skipping in the wild-type construct, an issue that can be mitigated by constructing the minigene with exons 2–9 (Fraile-Bethencourt et al. 2019). Following ACMG guidelines, BRCA2 c.475 + 5G > C can be categorized as a pathogenic variant (PVS1 + PS3_supporting + PM2 + PP3).

**Table 3:** Previous studies reporting the BRCA1 c.4358-2A > G variant

Num	Variant (cDNA)	Reported protein change	Nationality	Age	Cancer type	Family history of cancer	References
1	c.4358-2A > G	–	Armenian	40	OC&BC	Moth OC (50); Pat G. Moth LC; Sis OC	Moradian et al. (2021)
2	c.4358-2A > G	–	Chinese	55	OC	Sis OC (50); Bro RC (55)	Hu et al. (2022)
3	c.4358-2A > G	p. Ala1453Glyfs	Chinese	37	OC	Moth LC (50); Fath UC (71); Sis OC (41)	Current study
4	c.4358-2A > G	p. Ala1454Glyfs	Chinese	46	OC&BC	Fath EC (55)	Current study

*LC* lung cancer, *RC* rectal cancer, *EC* esophagus cancer, *UC* ureteral cancer, *Pat* paternal, *G* grand, *Moth* mother, *Fath* father; *Sis* sister, *Bro* brother

The therapeutic value of PARPi in OC patients with pathogenic/likely pathogenic BRCA variants has been widely recognized. The reclassification of BRCA1 c.4358-2A > G suggested a promising response to PARPi in carriers. However, patients A II:2 and A II:3 exhibited poorer response to PARPi. The lack of anticipated efficacy of PARPi within the family A has also spurred us to conduct a deeper discussion into whether the resistance mechanism of PARPi may be connected to the mutation site. Data from preclinical studies in mouse models suggest that different pathogenic variants may not all result in the same level of homologous recombination deficiency, which lead to primary PARPi resistance (Drost et al. 2011; Bouwman et al. 2013). Research on BRCA1 missense variants has demonstrated that the conserved N- and C-terminal domains play a crucial role in determining the response to therapies targeting homologous recombination deficiency (Bouwman et al. 2013). Mutated proteins that lose CtIP (C-terminal-binding protein 1 interacting protein) binding due to alterations in the BRCT domain can still support homologous recombination and confer resistance to PARPi, especially when they are present at high levels (Johnson et al. 2013). Reactivation of homologous recombination (HR) is recognized as one of the mechanisms of secondary PARPi resistance (Noordermeer and Attikum 2019). Reversion mutations of BRCA are reported to initiate HR reactivation, resulting in PARPi resistance (Barber et al. 2013; Edwards et al. 2008; Lheureux et al. 2017). Human pancreatic cancer cell lines resistant to PARPi demonstrated the expression of new BRCA2 isoforms resulting from the c.6174delT frameshift mutation. This mutation led to the restoration of the open reading frame of BRCA2, consequently reactivating the homologous recombination repair (Sakai et al. 2008). In addition, cells with tumor suppressor p53-binding protein 1 deficiency that retain a mutated BRCA1 protein with an intact coiled-coil (CC) domain––that is required for PALB2 binding––show increased reactivation of HR (Nacson et al. 2018). Therefore, it is crucial to further investigate and gather variant-specific information to understand the responses to PARPi more comprehensively (Bouwman and Jonkers 2014).

Currently, there is no internationally accepted standard for reporting BRCA tests, nor is there an agreed-upon classification system. Different laboratories employ varying approaches: some merely report variations without providing any interpretation, while others use narrative methods or rely on locally developed guidelines or published protocols (Moghadasi et al. 2013; Ryu et al. 2017). The majority of reports typically include only basic variant information, along with routine details such as age and family history. Nonetheless, given the widespread use of PARPi, it is essential to describe the therapeutic effects of these inhibitors on patients with different variants in a more informative manner. This would provide additional supporting information for research on drug resistance related to PARPi.

In addition to the OC patients, the variant BRCA1 c.4358-2A > G was also identified in the daughter (B III:1) of patient B II:2, leading to an elevated risk of cancer. Carriers of a pathogenic BRCA1 variant face an estimated 72% cumulative risk of BC by age 80 and a 48.3% cumulative risk of OC by age 70 (Kuchenbaecker et al. 2017; Chen et al. 2020). When it comes to BC screening, breast MRI is favored over mammography due to its heightened sensitivity. For the early screening of OC, although there is controversy, the combination of transvaginal ultrasound and serum CA-125 testing persists as the prevailing approach (Jacobs et al. 2016). The effectiveness of risk-reducing mastectomy (RRM) and risk-reducing salpingo-oophorectomy (RRSO) in reducing the risk of breast and ovarian cancer among carriers has been established through research (Rebbeck et al. 2009, 2004). However, the decision to undergo RRM or RRSO is a complex one, involving various considerations such as impacts on body image, psychological well-being and reproduction, as well as the potential risks associated with premature menopause (Honold and Camus 2018). Currently, the BRCA1 c.4358-2A > G carrier B III:3 is undergoing annual gynecological ultrasound, regular serum CA-125 testing, and periodic breast monitoring.

Our study also has several limitations. Firstly, it is a retrospective study conducted at a single center; therefore, the results may only reflect the regional incidence. A larger-scale investigation is required to determine the prevalence of VUS and intronic variants in the broader population. Additionally, the functional assays performed for the variant focused on the splicing changes. We only performed homologous modeling to analyze the structure of the mutated protein. Further investigations are necessary to determine the actual structure of the mutated protein and its potential impact on PARPi resistance.

## Conclusion

In accordance with the ACMG guidelines, we reclassify the BRCA1 c.4358-2A > G and BRCA2 c.475 + 5G > C as pathogenic variants based on minigene assays. The BRCA1 c.4358-2A > G might be one of the prevalent variants in the Chinese population. More research is needed to investigate the association between variants and PARPi resistance.

## Supplementary Information

Below is the link to the electronic supplementary material.

Supplementary file1 (DOCX 28 KB)

Supplementary file2 (DOCX 35 KB)

## Data Availability

The original data of the study can be obtained from the corresponding author on reasonable request.

## Abbreviations

ASCO: American Society of Clinical Oncology
BRCT: BRCA1 C-terminus
CtIP: C-terminal-binding protein 1 interacting protein
HBOC: Hereditary Breast and Ovarian Cancer
HR: Homologous Recombination
NCCN: National Comprehensive Cancer Network
OC: Ovarian Cancer
PARPi: Poly (ADP-ribose) Polymerase inhibitors
PBMCs: Peripheral Blood Mononuclear cells
RRM: Risk-Reducing Mastectomy
RRSO: Risk-Reducing Salpingo-Oophorectomy
VUS: Variants of Uncertain Significance

## References

[CR1] Abou Tayoun AN, Pesaran T, DiStefano MT et al (2018) Recommendations for interpreting the loss of function PVS1 ACMG/AMP variant criterion. Hum Mutat 39:1517–1524 30192042 10.1002/humu.23626 PMC6185798

[CR2] Barber LJ, Sandhu S, Chen L et al (2013) Secondary mutations in BRCA2 associated with clinical resistance to a PARP inhibitor. J Pathol 229:422–429 23165508 10.1002/path.4140

[CR3] Bienert S, Waterhouse A, de Beer TA et al (2017) The SWISS-MODEL repository-new features and functionality. Nucleic Acids Res 45:D313–D319 27899672 10.1093/nar/gkw1132 PMC5210589

[CR4] Bouwman P, Jonkers J (2014) Molecular pathways: how can BRCA-mutated tumors become resistant to PARP inhibitors? Clin Cancer Res 20:540–547 24270682 10.1158/1078-0432.CCR-13-0225

[CR5] Bouwman P, van der Gulden H, van der Heijden I et al (2013) A high-throughput functional complementation assay for classification of BRCA1 missense variants. Cancer Discov 3:1142–1155 23867111 10.1158/2159-8290.CD-13-0094

[CR6] Chen J, Bae E, Zhang L et al (2020) Penetrance of breast and ovarian cancer in women who carry a BRCA1/2 mutation and do not use risk-reducing salpingo-oophorectomy: an updated meta-analysis. JNCI Cancer Spectr 4:pkaa029 32676552 10.1093/jncics/pkaa029 PMC7353955

[CR7] Colombo M, Blok MJ, Whiley P et al (2014) Comprehensive annotation of splice junctions supports pervasive alternative splicing at the BRCA1 locus: a report from the ENIGMA consortium. Hum Mol Genet 23:3666–3680 24569164 10.1093/hmg/ddu075

[CR8] Domchek SM, Bradbury A, Garber JE et al (2013) Multiplex genetic testing for cancer susceptibility: out on the high wire without a net? J Clin Oncol 31:1267–1270 23460708 10.1200/JCO.2012.46.9403

[CR9] Drost R, Bouwman P, Rottenberg S et al (2011) BRCA1 RING function is essential for tumor suppression but dispensable for therapy resistance. Cancer Cell 20:797–809 22172724 10.1016/j.ccr.2011.11.014

[CR10] Easton DF, Steele L, Fields P et al (1997) Cancer risks in two large breast cancer families linked to BRCA2 on chromosome 13q12-13. Am J Hum Genet 61:120–128 9245992 10.1086/513891 PMC1715847

[CR11] Eccles DM, Mitchell G, Monteiro AN et al (2015) BRCA1 and BRCA2 genetic testing-pitfalls and recommendations for managing variants of uncertain clinical significance. Ann Oncol 26:2057–2065 26153499 10.1093/annonc/mdv278 PMC5006185

[CR12] Edwards SL, Brough R, Lord CJ et al (2008) Resistance to therapy caused by intragenic deletion in BRCA2. Nature 451:1111–1115 18264088 10.1038/nature06548

[CR13] Fanale D, Pivetti A, Cancelliere D et al (2022) BRCA1/2 variants of unknown significance in hereditary breast and ovarian cancer (HBOC) syndrome: looking for the hidden meaning. Crit Rev Oncol Hematol 172:103626 35150867 10.1016/j.critrevonc.2022.103626

[CR14] Finch AP, Lubinski J, Moller P et al (2014) Impact of oophorectomy on cancer incidence and mortality in women with a BRCA1 or BRCA2 mutation. J Clin Oncol 32:1547–1553 24567435 10.1200/JCO.2013.53.2820 PMC4026578

[CR15] Fraile-Bethencourt E, Valenzuela-Palomo A, Diez-Gomez B et al (2019) Mis-splicing in breast cancer: identification of pathogenic BRCA2 variants by systematic minigene assays. J Pathol 248:409–420 30883759 10.1002/path.5268

[CR16] Gonzalez-Martin A, Pothuri B, Vergote I et al (2019) Niraparib in patients with newly diagnosed advanced ovarian cancer. N Engl J Med 381:2391–2402 31562799 10.1056/NEJMoa1910962

[CR17] Hoberg-Vetti H, Ognedal E, Buisson A et al (2020) The intronic BRCA1 c.5407–25T>A variant causing partly skipping of exon 23-a likely pathogenic variant with reduced penetrance? Eur J Hum Genet 28:1078–1086 32203205 10.1038/s41431-020-0612-1 PMC7382492

[CR18] Honold F, Camus M (2018) Prophylactic mastectomy versus surveillance for the prevention of breast cancer in women’s BRCA carriers. Medwave 18:e7161 30052622 10.5867/medwave.2018.04.7160

[CR19] Hu PZ, Chen XY, Xiong W et al (2022) A BRCA1 splice site variant responsible for familial ovarian cancer in a Han–Chinese family. Curr Med Sci 42:666–672 35290602 10.1007/s11596-022-2527-2

[CR20] Jacobs IJ, Menon U, Ryan A et al (2016) Ovarian cancer screening and mortality in the UK collaborative trial of ovarian cancer screening (UKCTOCS): a randomised controlled trial. Lancet 387:945–956 26707054 10.1016/S0140-6736(15)01224-6 PMC4779792

[CR21] Jaganathan K, Kyriazopoulou Panagiotopoulou S, McRae JF et al (2019) Predicting splicing from primary sequence with deep learning. Cell 176:535.e24-548.e24 30661751 10.1016/j.cell.2018.12.015

[CR22] Jimenez-Sainz J, Jensen RB (2021) Imprecise medicine: BRCA2 variants of uncertain significance (VUS), the challenges and benefits to integrate a functional assay workflow with clinical decision rules. Genes (basel) 12(5):780 34065235 10.3390/genes12050780 PMC8161351

[CR23] Johnson N, Johnson SF, Yao W et al (2013) Stabilization of mutant BRCA1 protein confers PARP inhibitor and platinum resistance. Proc Natl Acad Sci U S A 110:17041–17046 24085845 10.1073/pnas.1305170110 PMC3801063

[CR24] Jumper J, Evans R, Pritzel A et al (2021) Highly accurate protein structure prediction with AlphaFold. Nature 596:583–589 34265844 10.1038/s41586-021-03819-2 PMC8371605

[CR25] Kuchenbaecker KB, Hopper JL, Barnes DR et al (2017) Risks of breast, ovarian, and contralateral breast cancer for BRCA1 and BRCA2 mutation carriers. JAMA 317:2402–2416 28632866 10.1001/jama.2017.7112

[CR26] Lheureux S, Bruce JP, Burnier JV et al (2017) Somatic BRCA1/2 recovery as a resistance mechanism after exceptional response to poly (ADP-ribose) polymerase inhibition. J Clin Oncol 35:1240–1249 28221868 10.1200/JCO.2016.71.3677

[CR27] Li Q, Wang K (2017) InterVar: clinical interpretation of genetic variants by the 2015 ACMG-AMP guidelines. Am J Hum Genet 100:267–280 28132688 10.1016/j.ajhg.2017.01.004 PMC5294755

[CR28] Miki Y, Swensen J, Shattuck-Eidens D et al (1994) A strong candidate for the breast and ovarian cancer susceptibility gene BRCA1. Science 266:66–71 7545954 10.1126/science.7545954

[CR29] Moghadasi S, Hofland N, Wouts JN et al (2013) Variants of uncertain significance in BRCA1 and BRCA2 assessment of in silico analysis and a proposal for communication in genetic counselling. J Med Genet 50:74–79 23231788 10.1136/jmedgenet-2012-100961

[CR30] Moore K, Colombo N, Scambia G et al (2018) Maintenance olaparib in patients with newly diagnosed advanced ovarian cancer. N Engl J Med 379:2495–2505 30345884 10.1056/NEJMoa1810858

[CR31] Moradian MM, Babikyan DT, Markarian S et al (2021) Germline mutational spectrum in Armenian breast cancer patients suspected of hereditary breast and ovarian cancer. Hum Genome Var 8:9 33558524 10.1038/s41439-021-00140-2 PMC7870655

[CR32] Nacson J, Krais JJ, Bernhardy AJ et al (2018) BRCA1 mutation-specific responses to 53BP1 loss-induced homologous recombination and PARP inhibitor resistance. Cell Rep 25:1384 30380426 10.1016/j.celrep.2018.10.009 PMC6296467

[CR33] Noordermeer SM, van Attikum H (2019) PARP inhibitor resistance: a tug-of-war in BRCA-mutated cells. Trends Cell Biol 29:820–834 31421928 10.1016/j.tcb.2019.07.008

[CR34] Perrin-Vidoz L, Sinilnikova OM, Stoppa-Lyonnet D et al (2002) The nonsense-mediated mRNA decay pathway triggers degradation of most BRCA1 mRNAs bearing premature termination codons. Hum Mol Genet 11:2805–2814 12393792 10.1093/hmg/11.23.2805

[CR35] Ponti G, De Angelis C, Ponti R et al (2023) Hereditary breast and ovarian cancer: from genes to molecular targeted therapies. Crit Rev Clin Lab Sci 60:640–650 37455374 10.1080/10408363.2023.2234488

[CR36] Rebbeck TR, Friebel T, Lynch HT et al (2004) Bilateral prophylactic mastectomy reduces breast cancer risk in BRCA1 and BRCA2 mutation carriers: the PROSE study group. J Clin Oncol 22:1055–1062 14981104 10.1200/JCO.2004.04.188

[CR37] Rebbeck TR, Kauff ND, Domchek SM (2009) Meta-analysis of risk reduction estimates associated with risk-reducing salpingo-oophorectomy in BRCA1 or BRCA2 mutation carriers. J Natl Cancer Inst 101:80–87 19141781 10.1093/jnci/djn442 PMC2639318

[CR38] Reese MG, Eeckman FH, Kulp D et al (1997) Improved splice site detection in genie. J Comput Biol 4:311–323 9278062 10.1089/cmb.1997.4.311

[CR39] Reuter P, Walter M, Kohl S et al (2023) Systematic analysis of CNGA3 splice variants identifies different mechanisms of aberrant splicing. Sci Rep 13:2896 36801918 10.1038/s41598-023-29452-9 PMC9938885

[CR40] Richards S, Aziz N, Bale S et al (2015) Standards and guidelines for the interpretation of sequence variants: a joint consensus recommendation of the American college of medical genetics and genomics and the association for molecular pathology. Genet Med 17:405–424 25741868 10.1038/gim.2015.30 PMC4544753

[CR41] Ryu JM, Kang G, Nam SJ et al (2017) Suggestion of BRCA1 c.5339T>C (p.L1780P) variant confer from “unknown significance” to “Likely pathogenic” based on clinical evidence in Korea. Breast 33:109–116 28364669 10.1016/j.breast.2017.03.006

[CR42] Sakai W, Swisher EM, Karlan BY et al (2008) Secondary mutations as a mechanism of cisplatin resistance in BRCA2-mutated cancers. Nature 451:1116–1120 18264087 10.1038/nature06633 PMC2577037

[CR43] Sanz DJ, Acedo A, Infante M et al (2010) A high proportion of DNA variants of BRCA1 and BRCA2 is associated with aberrant splicing in breast/ovarian cancer patients. Clin Cancer Res 16:1957–1967 20215541 10.1158/1078-0432.CCR-09-2564

[CR44] Tew WP, Lacchetti C, Ellis A et al (2020) PARP inhibitors in the management of ovarian cancer: ASCO guideline. J Clin Oncol 38:3468–3493 32790492 10.1200/JCO.20.01924 PMC8942301

[CR45] Valenzuela-Palomo A, Bueno-Martinez E, Sanoguera-Miralles L et al (2022) Splicing predictions, minigene analyses, and ACMG-AMP clinical classification of 42 germline PALB2 splice-site variants. J Pathol 256:321–334 34846068 10.1002/path.5839 PMC9306493

[CR46] Varadi M, Anyango S, Deshpande M et al (2022) AlphaFold protein structure database: massively expanding the structural coverage of protein-sequence space with high-accuracy models. Nucleic Acids Res 50:D439–D444 34791371 10.1093/nar/gkab1061 PMC8728224

[CR47] Wang GS, Cooper TA (2007) Splicing in disease: disruption of the splicing code and the decoding machinery. Nat Rev Genet 8:749–761 17726481 10.1038/nrg2164

[CR48] Waterhouse A, Bertoni M, Bienert S et al (2018) SWISS-MODEL: homology modelling of protein structures and complexes. Nucleic Acids Res 46:W296–W303 29788355 10.1093/nar/gky427 PMC6030848

[CR49] Weisschuh N, Wissinger B, Gramer E (2012) A splice site mutation in the PAX6 gene which induces exon skipping causes autosomal dominant inherited aniridia. Mol vis 18:751–757 22509105 PMC3324353

[CR50] Wooster R, Bignell G, Lancaster J et al (1995) Identification of the breast cancer susceptibility gene BRCA2. Nature 378:789–792 8524414 10.1038/378789a0

[CR51] Yeo G, Burge CB (2004) Maximum entropy modeling of short sequence motifs with applications to RNA splicing signals. J Comput Biol 11:377–394 15285897 10.1089/1066527041410418

