# Supplementary material for: Reclassifying BRCA1 c.4358-2A > G and BRCA2 c.475 + 5G > C variants from “Uncertain Significance” to “Pathogenic” based on minigene assays and clinical evidence
Source: J Cancer Res Clin Oncol. 2024 Feb 1;150(2):62. doi: 10.1007/s00432-023-05597-y (PMC10834553; doi:10.1007/s00432-023-05597-y)
Supplement: Supplementary file 1 — Supplementary file1 (DOCX 28 KB) [file 432_2023_5597_MOESM1_ESM.docx]

| **STable 1 Primer Sequences** | | |
| --- | --- | --- |
| Name |  | Primer Sequence (5'-3') |
| Sequence amplification primers | F | gatcccagatatctggtgatTTATTATTTTTTAATCATTGAATTCCATTT |
|  | R | tccttccacacaggtacgggGCACATTTCTCATGTTGTAGCTTATG |
| BRCA1 c.4358-2 point mutation primer | F | CATTCCATTTAAgGCAGTATTAACTTCACAGAAAAGTAGTGA |
|  | R | CTGCcTTAAATGGAATGAGAAAACAAATCTACTT |

| **STable 2 Identified VUS of BRCA1** | | | | | |
| --- | --- | --- | --- | --- | --- |
| cDNA change | Frequence | Exon | Protien change | ClinVar | GnomAD (Allele Frequency) |
| c.71G>T | 1 | 2 | p.Cys24Phe | Not provided​ | None |
| c.343C>A | 1 | 5 | p.Pro115Thr | VUS | 0.0004% |
| c.446A>C | 1 | 7 | p.Glu149Ala | Conflicting | 0.0050% |
| c.449C>T | 1 | 7 | p.Thr150Ile | **Novel** | None |
| c.763G>C | 1 | 10 | p.Glu255Gln | **Novel** | None |
| c.1202G>T | 1 | 10 | p.Gly401Val | **Novel** | None |
| c.1367T>C | 1 | 10 | p.Ile456Thr | Conflicting | 0.0008% |
| c.1427A>T | 1 | 10 | p.His476Leu | **Novel** | None |
| c.2481A>C | 1 | 10 | p.Glu827Asp | Conflicting | 0.0004% |
| c.2611C>A | 1 | 10 | p.Pro871Thr | **Novel** | None |
| c.2933A>G | 2 | 10 | p.Tyr978Cys | Conflicting | 0.0004% |
| c.3083G>C | 1 | 10 | p.Arg1028Pro | **Novel** | None |
| c.3287A>G | 1 | 10 | p.Gln1096Arg | Conflicting | None |
| c.3466G>A | 1 | 10 | p.Asp1156Asn | Conflicting | None |
| c.4000G>T | 1 | 10 | p.Gly1334Cys | **Novel** | None |
| c.4060_4071del | 1 | 10 | p.Asn1354_Glu1357del | **Novel** | None |
| c.4075C>G | 1 | 10 | p.Gln1359Glu | Conflicting | None |
| c.4293C>G | 1 | 12 | p.Ser1431Asp | **Novel** | None |
| c.4358-2A>G | 2 | Intron12 | - | VUS | None |
| c.4967G>A | 1 | 15 | p.Gly1656Asp | VUS | None |
| c.5066T>C | 1 | 16 | p.Met1689Thr | VUS | None |

| **STable 3 Identified VUS of BRCA2** | | | | | |
| --- | --- | --- | --- | --- | --- |
| cDNA change | Frequence | Exon | Protien change | ClinVar | GnomAD (Allele Frequency) |
| c.14C>A | 1 | 2 | p.Ser5Tyr | **Novel** | None |
| c.112G>A | 1 | 3 | p.Glu38Lys | VUS | None |
| c.241T>G | 1 | 11 | p.Phe81Val | VUS | 0.0008% |
| c.475+5G>C | 1 | intron5 | - | **Novel** | None |
| c.983C>T | 1 | 10 | p.Thr328Ile | **Novel** | None |
| c.1211A>T | 1 | 10 | p.Asn404Ile | Conflicting | 0.0004% |
| c.1370A>C | 1 | 10 | p.Lys457Thr | **Novel** | None |
| c.1382A>C | 1 | 10 | p.Glu461Ala | Conflicting | None |
| c.1909+22delT | 1 | intron10 | - | Conflicting | None |
| c.2186T>C | 1 | 11 | p.Ile729Thr | Conflicting | 0.0016% |
| c.2347G>A | 1 | 11 | p.Val783Ile | Conflicting | None |
| c.2444T>C | 1 | 11 | p.Met815Thr | Conflicting | None |
| c.2946A>G | 1 | 11 | p.Ile982Met | Conflicting | 0.0088% |
| c.3671G>T | 1 | 11 | p.Gly1224Val | Conflicting | None |
| c.3794G>T | 1 | 11 | p.Cys1265Phe | Conflicting | 0.0032% |
| c.3803C>A | 1 | 11 | p. Ser1268Tyr | **Novel** | None |
| c.4376A>G | 1 | 11 | p.Asn1459Ser） | Conflicting | 0.0043% |
| c.4559C>G | 1 | 11 | p.Thr1520Ser | Conflicting | 0.0004% |
| c.4785G>T | 1 | 11 | p.Gln1595His | Conflicting | None |
| c.5501G>T | 1 | 11 | p.Ser1834Ile | **Novel** | None |
| c.5683G>A | 1 | 11 | p.Glu1895Lys | Conflicting | 0.0011% |
| c.5893C>T | 1 | 11 | p.Leu1965Phe | Conflicting | 0.0020% |
| c.5901G>T | 1 | 11 | p.K1967NLys1967Asn | **Novel** | None |
| c.6093T>A | 1 | 11 | p.Thr2031= | **Novel** | None |
| c.6710A>G | 1 | 11 | p.Asp2237Gly | Conflicting | None |
| c.7013C>T | 1 | 14 | p.Thr2338Ile | **Novel** | None |
| c.7022G>A | 1 | 14 | p.Arg2341His | VUS ​ | None |
| c.7088A>G | 2 | 14 | p.Tyr2363Cys | VUS | 0.0004% |
| c.7507G>A | 1 | 15 | p.Val2503Ile | Conflicting | 0.0004% |
| c.7618-15_7618-14del | 1 | intron15 | - | **Novel** | None |
| c.7926T>A | 1 | 17 | p.Phe2642Leu | **Novel** | None |
| c.8284C>G | 1 | 18 | p.Pro2762Ala | **Novel** | None |
| c.8299C>T | 1 | 18 | p.Pro2767Ser | Conflicting | None |
| c.8382C>A | 3 | 19 | p.F2794LPhe2794Leu | **Novel** | None |
| c.8474C>A | 1 | 19 | p.Ala2825Glu | VUS | None |
| c.8481T>G | 1 | 19 | p.Pro2827= | **Novel** | None |
| c.8657C>A | 1 | 21 | p. Pro2886Gln | **Novel** | None |
| c.8971C>T | 2 | 23 | p.Arg2991Cys | Conflicting | 0.0008% |
| c.9011A>G | 1 | 23 | p.Lys3004Arg | Conflicting | None |
| c.9116C>T | 1 | 23 | p.Pro3039Leu | Conflicting | 0.0089% |
| c.9335A>G | 1 | 25 | p.Asp3112Gly | VUS | None |
| c.9626C>A | 1 | 26 | p.Pro3209His | **Novel** | None |
| c.9959C>T | 1 | 27 | p.Pro3320Leu | VUS | None |
| c.9983T>G | 1 | 27 | p.Phe3328Cys | VUS | 0.0004% |
| c.10054C>G | 1 | 27 | p.Leu3352Val | VUS | 0.0004% |
| c.10131A>C | 1 | 27 | p.Glu3377Asp | VUS | None |
