# Supplementary material for: Reclassifying BRCA1 c.4358-2A > G and BRCA2 c.475 + 5G > C variants from “Uncertain Significance” to “Pathogenic” based on minigene assays and clinical evidence
Source: J Cancer Res Clin Oncol. 2024 Feb 1;150(2):62. doi: 10.1007/s00432-023-05597-y (PMC10834553; doi:10.1007/s00432-023-05597-y)
Supplement: Supplementary file 2 — Supplementary file2 (DOCX 35 KB) [file 432_2023_5597_MOESM2_ESM.docx]

**BRCA1 [ Homo sapiens (human) ]**

Full name: BRCA1

Transcript ID: NM_007294.4

Mutation: c.4358-2A>G

Chromosome: 17

**>BRCA1 WT Nucleotide Sequence (5592 nt):**

ATGGATTTATCTGCTCTTCGCGTTGAAGAAGTACAAAATGTCATTAATGCTATGCAGAAAATCTTAGAGTGTCCCATCTGTCTGGAGTTGATCAAGGAACCTGTCTCCACAAAGTGTGACCACATATTTTGCAAATTTTGCATGCTGAAACTTCTCAACCAGAAGAAAGGGCCTTCACAGTGTCCTTTATGTAAGAATGATATAACCAAAAGGAGCCTACAAGAAAGTACGAGATTTAGTCAACTTGTTGAAGAGCTATTGAAAATCATTTGTGCTTTTCAGCTTGACACAGGTTTGGAGTATGCAAACAGCTATAATTTTGCAAAAAAGGAAAATAACTCTCCTGAACATCTAAAAGATGAAGTTTCTATCATCCAAAGTATGGGCTACAGAAACCGTGCCAAAAGACTTCTACAGAGTGAACCCGAAAATCCTTCCTTGCAGGAAACCAGTCTCAGTGTCCAACTCTCTAACCTTGGAACTGTGAGAACTCTGAGGACAAAGCAGCGGATACAACCTCAAAAGACGTCTGTCTACATTGAATTGGGATCTGATTCTTCTGAAGATACCGTTAATAAGGCAACTTATTGCAGTGTGGGAGATCAAGAATTGTTACAAATCACCCCTCAAGGAACCAGGGATGAAATCAGTTTGGATTCTGCAAAAAAGGCTGCTTGTGAATTTTCTGAGACGGATGTAACAAATACTGAACATCATCAACCCAGTAATAATGATTTGAACACCACTGAGAAGCGTGCAGCTGAGAGGCATCCAGAAAAGTATCAGGGTAGTTCTGTTTCAAACTTGCATGTGGAGCCATGTGGCACAAATACTCATGCCAGCTCATTACAGCATGAGAACAGCAGTTTATTACTCACTAAAGACAGAATGAATGTAGAAAAGGCTGAATTCTGTAATAAAAGCAAACAGCCTGGCTTAGCAAGGAGCCAACATAACAGATGGGCTGGAAGTAAGGAAACATGTAATGATAGGCGGACTCCCAGCACAGAAAAAAAGGTAGATCTGAATGCTGATCCCCTGTGTGAGAGAAAAGAATGGAATAAGCAGAAACTGCCATGCTCAGAGAATCCTAGAGATACTGAAGATGTTCCTTGGATAACACTAAATAGCAGCATTCAGAAAGTTAATGAGTGGTTTTCCAGAAGTGATGAACTGTTAGGTTCTGATGACTCACATGATGGGGAGTCTGAATCAAATGCCAAAGTAGCTGATGTATTGGACGTTCTAAATGAGGTAGATGAATATTCTGGTTCTTCAGAGAAAATAGACTTACTGGCCAGTGATCCTCATGAGGCTTTAATATGTAAAAGTGAAAGAGTTCACTCCAAATCAGTAGAGAGTAATATTGAAGACAAAATATTTGGGAAAACCTATCGGAAGAAGGCAAGCCTCCCCAACTTAAGCCATGTAACTGAAAATCTAATTATAGGAGCATTTGTTACTGAGCCACAGATAATACAAGAGCGTCCCCTCACAAATAAATTAAAGCGTAAAAGGAGACCTACATCAGGCCTTCATCCTGAGGATTTTATCAAGAAAGCAGATTTGGCAGTTCAAAAGACTCCTGAAATGATAAATCAGGGAACTAACCAAACGGAGCAGAATGGTCAAGTGATGAATATTACTAATAGTGGTCATGAGAATAAAACAAAAGGTGATTCTATTCAGAATGAGAAAAATCCTAACCCAATAGAATCACTCGAAAAAGAATCTGCTTTCAAAACGAAAGCTGAACCTATAAGCAGCAGTATAAGCAATATGGAACTCGAATTAAATATCCACAATTCAAAAGCACCTAAAAAGAATAGGCTGAGGAGGAAGTCTTCTACCAGGCATATTCATGCGCTTGAACTAGTAGTCAGTAGAAATCTAAGCCCACCTAATTGTACTGAATTGCAAATTGATAGTTGTTCTAGCAGTGAAGAGATAAAGAAAAAAAAGTACAACCAAATGCCAGTCAGGCACAGCAGAAACCTACAACTCATGGAAGGTAAAGAACCTGCAACTGGAGCCAAGAAGAGTAACAAGCCAAATGAACAGACAAGTAAAAGACATGACAGCGATACTTTCCCAGAGCTGAAGTTAACAAATGCACCTGGTTCTTTTACTAAGTGTTCAAATACCAGTGAACTTAAAGAATTTGTCAATCCTAGCCTTCCAAGAGAAGAAAAAGAAGAGAAACTAGAAACAGTTAAAGTGTCTAATAATGCTGAAGACCCCAAAGATCTCATGTTAAGTGGAGAAAGGGTTTTGCAAACTGAAAGATCTGTAGAGAGTAGCAGTATTTCATTGGTACCTGGTACTGATTATGGCACTCAGGAAAGTATCTCGTTACTGGAAGTTAGCACTCTAGGGAAGGCAAAAACAGAACCAAATAAATGTGTGAGTCAGTGTGCAGCATTTGAAAACCCCAAGGGACTAATTCATGGTTGTTCCAAAGATAATAGAAATGACACAGAAGGCTTTAAGTATCCATTGGGACATGAAGTTAACCACAGTCGGGAAACAAGCATAGAAATGGAAGAAAGTGAACTTGATGCTCAGTATTTGCAGAATACATTCAAGGTTTCAAAGCGCCAGTCATTTGCTCCGTTTTCAAATCCAGGAAATGCAGAAGAGGAATGTGCAACATTCTCTGCCCACTCTGGGTCCTTAAAGAAACAAAGTCCAAAAGTCACTTTTGAATGTGAACAAAAGGAAGAAAATCAAGGAAAGAATGAGTCTAATATCAAGCCTGTACAGACAGTTAATATCACTGCAGGCTTTCCTGTGGTTGGTCAGAAAGATAAGCCAGTTGATAATGCCAAATGTAGTATCAAAGGAGGCTCTAGGTTTTGTCTATCATCTCAGTTCAGAGGCAACGAAACTGGACTCATTACTCCAAATAAACATGGACTTTTACAAAACCCATATCGTATACCACCACTTTTTCCCATCAAGTCATTTGTTAAAACTAAATGTAAGAAAAATCTGCTAGAGGAAAACTTTGAGGAACATTCAATGTCACCTGAAAGAGAAATGGGAAATGAGAACATTCCAAGTACAGTGAGCACAATTAGCCGTAATAACATTAGAGAAAATGTTTTTAAAGAAGCCAGCTCAAGCAATATTAATGAAGTAGGTTCCAGTACTAATGAAGTGGGCTCCAGTATTAATGAAATAGGTTCCAGTGATGAAAACATTCAAGCAGAACTAGGTAGAAACAGAGGGCCAAAATTGAATGCTATGCTTAGATTAGGGGTTTTGCAACCTGAGGTCTATAAACAAAGTCTTCCTGGAAGTAATTGTAAGCATCCTGAAATAAAAAAGCAAGAATATGAAGAAGTAGTTCAGACTGTTAATACAGATTTCTCTCCATATCTGATTTCAGATAACTTAGAACAGCCTATGGGAAGTAGTCATGCATCTCAGGTTTGTTCTGAGACACCTGATGACCTGTTAGATGATGGTGAAATAAAGGAAGATACTAGTTTTGCTGAAAATGACATTAAGGAAAGTTCTGCTGTTTTTAGCAAAAGCGTCCAGAAAGGAGAGCTTAGCAGGAGTCCTAGCCCTTTCACCCATACACATTTGGCTCAGGGTTACCGAAGAGGGGCCAAGAAATTAGAGTCCTCAGAAGAGAACTTATCTAGTGAGGATGAAGAGCTTCCCTGCTTCCAACACTTGTTATTTGGTAAAGTAAACAATATACCTTCTCAGTCTACTAGGCATAGCACCGTTGCTACCGAGTGTCTGTCTAAGAACACAGAGGAGAATTTATTATCATTGAAGAATAGCTTAAATGACTGCAGTAACCAGGTAATATTGGCAAAGGCATCTCAGGAACATCACCTTAGTGAGGAAACAAAATGTTCTGCTAGCTTGTTTTCTTCACAGTGCAGTGAATTGGAAGACTTGACTGCAAATACAAACACCCAGGATCCTTTCTTGATTGGTTCTTCCAAACAAATGAGGCATCAGTCTGAAAGCCAGGGAGTTGGTCTGAGTGACAAGGAATTGGTTTCAGATGATGAAGAAAGAGGAACGGGCTTGGAAGAAAATAATCAAGAAGAGCAAAGCATGGATTCAAACTTAGGTGAAGCAGCATCTGGGTGTGAGAGTGAAACAAGCGTCTCTGAAGACTGCTCAGGGCTATCCTCTCAGAGTGACATTTTAACCACTCAGCAGAGGGATACCATGCAACATAACCTGATAAAGCTCCAGCAGGAAATGGCTGAACTAGAAGCTGTGTTAGAACAGCATGGGAGCCAGCCTTCTAACAGCTACCCTTCCATCATAAGTGACTCTTCTGCCCTTGAGGACCTGCGAAATCCAGAACAAAGCACATCAGAAAAA**GCA**GTATTAACTTCACAGAAAAGTAGTGAATACCCTATAAGCCAGAATCCAGAAGGCCTTTCTGCTGACAAGTTTGAGGTGTCTGCAGATAGTTCTACCAGTAAAAATAAAGAACCAGGAGTGGAAAGGTCATCCCCTTCTAAATGCCCATCATTAGATGATAGGTGGTACATGCACAGTTGCTCTGGGAGTCTTCAGAATAGAAACTACCCATCTCAAGAGGAGCTCATTAAGGTTGTTGATGTGGAGGAGCAACAGCTGGAAGAGTCTGGGCCACACGATTTGACGGAAACATCTTACTTGCCAAGGCAAGATCTAGAGGGAACCCCTTACCTGGAATCTGGAATCAGCCTCTTCTCTGATGACCCTGAATCTGATCCTTCTGAAGACAGAGCCCCAGAGTCAGCTCGTGTTGGCAACATACCATCTTCAACCTCTGCATTGAAAGTTCCCCAATTGAAAGTTGCAGAATCTGCCCAGAGTCCAGCTGCTGCTCATACTACTGATACTGCTGGGTATAATGCAATGGAAGAAAGTGTGAGCAGGGAGAAGCCAGAATTGACAGCTTCAACAGAAAGGGTCAACAAAAGAATGTCCATGGTGGTGTCTGGCCTGACCCCAGAAGAATTTATGCTCGTGTACAAGTTTGCCAGAAAACACCACATCACTTTAACTAATCTAATTACTGAAGAGACTACTCATGTTGTTATGAAAACAGATGCTGAGTTTGTGTGTGAACGGACACTGAAATATTTTCTAGGAATTGCGGGAGGAAAATGGGTAGTTAGCTATTTCTGGGTGACCCAGTCTATTAAAGAAAGAAAAATGCTGAATGAGCATGATTTTGAAGTCAGAGGAGATGTGGTCAATGGAAGAAACCACCAAGGTCCAAAGCGAGCAAGAGAATCCCAGGACAGAAAGATCTTCAGGGGGCTAGAAATCTGTTGCTATGGGCCCTTCACCAACATGCCCACAGATCAACTGGAATGGATGGTACAGCTGTGTGGTGCTTCTGTGGTGAAGGAGCTTTCATCATTCACCCTTGGCACAGGTGTCCACCCAATTGTGGTTGTGCAGCCAGATGCCTGGACAGAGGACAATGGCTTCCATGCAATTGGGCAGATGTGTGAGGCACCTGTGGTGACCCGAGAGTGGGTGTTGGACAGTGTAGCACTCTACCAGTGCCAGGAGCTGGACACCTACCTGATACCCCAGATCCCCCACAGCCACTACTGA

**>BRCA1 c.4358-2A>G Nucleotide Sequence (5592 nt)**

ATGGATTTATCTGCTCTTCGCGTTGAAGAAGTACAAAATGTCATTAATGCTATGCAGAAAATCTTAGAGTGTCCCATCTGTCTGGAGTTGATCAAGGAACCTGTCTCCACAAAGTGTGACCACATATTTTGCAAATTTTGCATGCTGAAACTTCTCAACCAGAAGAAAGGGCCTTCACAGTGTCCTTTATGTAAGAATGATATAACCAAAAGGAGCCTACAAGAAAGTACGAGATTTAGTCAACTTGTTGAAGAGCTATTGAAAATCATTTGTGCTTTTCAGCTTGACACAGGTTTGGAGTATGCAAACAGCTATAATTTTGCAAAAAAGGAAAATAACTCTCCTGAACATCTAAAAGATGAAGTTTCTATCATCCAAAGTATGGGCTACAGAAACCGTGCCAAAAGACTTCTACAGAGTGAACCCGAAAATCCTTCCTTGCAGGAAACCAGTCTCAGTGTCCAACTCTCTAACCTTGGAACTGTGAGAACTCTGAGGACAAAGCAGCGGATACAACCTCAAAAGACGTCTGTCTACATTGAATTGGGATCTGATTCTTCTGAAGATACCGTTAATAAGGCAACTTATTGCAGTGTGGGAGATCAAGAATTGTTACAAATCACCCCTCAAGGAACCAGGGATGAAATCAGTTTGGATTCTGCAAAAAAGGCTGCTTGTGAATTTTCTGAGACGGATGTAACAAATACTGAACATCATCAACCCAGTAATAATGATTTGAACACCACTGAGAAGCGTGCAGCTGAGAGGCATCCAGAAAAGTATCAGGGTAGTTCTGTTTCAAACTTGCATGTGGAGCCATGTGGCACAAATACTCATGCCAGCTCATTACAGCATGAGAACAGCAGTTTATTACTCACTAAAGACAGAATGAATGTAGAAAAGGCTGAATTCTGTAATAAAAGCAAACAGCCTGGCTTAGCAAGGAGCCAACATAACAGATGGGCTGGAAGTAAGGAAACATGTAATGATAGGCGGACTCCCAGCACAGAAAAAAAGGTAGATCTGAATGCTGATCCCCTGTGTGAGAGAAAAGAATGGAATAAGCAGAAACTGCCATGCTCAGAGAATCCTAGAGATACTGAAGATGTTCCTTGGATAACACTAAATAGCAGCATTCAGAAAGTTAATGAGTGGTTTTCCAGAAGTGATGAACTGTTAGGTTCTGATGACTCACATGATGGGGAGTCTGAATCAAATGCCAAAGTAGCTGATGTATTGGACGTTCTAAATGAGGTAGATGAATATTCTGGTTCTTCAGAGAAAATAGACTTACTGGCCAGTGATCCTCATGAGGCTTTAATATGTAAAAGTGAAAGAGTTCACTCCAAATCAGTAGAGAGTAATATTGAAGACAAAATATTTGGGAAAACCTATCGGAAGAAGGCAAGCCTCCCCAACTTAAGCCATGTAACTGAAAATCTAATTATAGGAGCATTTGTTACTGAGCCACAGATAATACAAGAGCGTCCCCTCACAAATAAATTAAAGCGTAAAAGGAGACCTACATCAGGCCTTCATCCTGAGGATTTTATCAAGAAAGCAGATTTGGCAGTTCAAAAGACTCCTGAAATGATAAATCAGGGAACTAACCAAACGGAGCAGAATGGTCAAGTGATGAATATTACTAATAGTGGTCATGAGAATAAAACAAAAGGTGATTCTATTCAGAATGAGAAAAATCCTAACCCAATAGAATCACTCGAAAAAGAATCTGCTTTCAAAACGAAAGCTGAACCTATAAGCAGCAGTATAAGCAATATGGAACTCGAATTAAATATCCACAATTCAAAAGCACCTAAAAAGAATAGGCTGAGGAGGAAGTCTTCTACCAGGCATATTCATGCGCTTGAACTAGTAGTCAGTAGAAATCTAAGCCCACCTAATTGTACTGAATTGCAAATTGATAGTTGTTCTAGCAGTGAAGAGATAAAGAAAAAAAAGTACAACCAAATGCCAGTCAGGCACAGCAGAAACCTACAACTCATGGAAGGTAAAGAACCTGCAACTGGAGCCAAGAAGAGTAACAAGCCAAATGAACAGACAAGTAAAAGACATGACAGCGATACTTTCCCAGAGCTGAAGTTAACAAATGCACCTGGTTCTTTTACTAAGTGTTCAAATACCAGTGAACTTAAAGAATTTGTCAATCCTAGCCTTCCAAGAGAAGAAAAAGAAGAGAAACTAGAAACAGTTAAAGTGTCTAATAATGCTGAAGACCCCAAAGATCTCATGTTAAGTGGAGAAAGGGTTTTGCAAACTGAAAGATCTGTAGAGAGTAGCAGTATTTCATTGGTACCTGGTACTGATTATGGCACTCAGGAAAGTATCTCGTTACTGGAAGTTAGCACTCTAGGGAAGGCAAAAACAGAACCAAATAAATGTGTGAGTCAGTGTGCAGCATTTGAAAACCCCAAGGGACTAATTCATGGTTGTTCCAAAGATAATAGAAATGACACAGAAGGCTTTAAGTATCCATTGGGACATGAAGTTAACCACAGTCGGGAAACAAGCATAGAAATGGAAGAAAGTGAACTTGATGCTCAGTATTTGCAGAATACATTCAAGGTTTCAAAGCGCCAGTCATTTGCTCCGTTTTCAAATCCAGGAAATGCAGAAGAGGAATGTGCAACATTCTCTGCCCACTCTGGGTCCTTAAAGAAACAAAGTCCAAAAGTCACTTTTGAATGTGAACAAAAGGAAGAAAATCAAGGAAAGAATGAGTCTAATATCAAGCCTGTACAGACAGTTAATATCACTGCAGGCTTTCCTGTGGTTGGTCAGAAAGATAAGCCAGTTGATAATGCCAAATGTAGTATCAAAGGAGGCTCTAGGTTTTGTCTATCATCTCAGTTCAGAGGCAACGAAACTGGACTCATTACTCCAAATAAACATGGACTTTTACAAAACCCATATCGTATACCACCACTTTTTCCCATCAAGTCATTTGTTAAAACTAAATGTAAGAAAAATCTGCTAGAGGAAAACTTTGAGGAACATTCAATGTCACCTGAAAGAGAAATGGGAAATGAGAACATTCCAAGTACAGTGAGCACAATTAGCCGTAATAACATTAGAGAAAATGTTTTTAAAGAAGCCAGCTCAAGCAATATTAATGAAGTAGGTTCCAGTACTAATGAAGTGGGCTCCAGTATTAATGAAATAGGTTCCAGTGATGAAAACATTCAAGCAGAACTAGGTAGAAACAGAGGGCCAAAATTGAATGCTATGCTTAGATTAGGGGTTTTGCAACCTGAGGTCTATAAACAAAGTCTTCCTGGAAGTAATTGTAAGCATCCTGAAATAAAAAAGCAAGAATATGAAGAAGTAGTTCAGACTGTTAATACAGATTTCTCTCCATATCTGATTTCAGATAACTTAGAACAGCCTATGGGAAGTAGTCATGCATCTCAGGTTTGTTCTGAGACACCTGATGACCTGTTAGATGATGGTGAAATAAAGGAAGATACTAGTTTTGCTGAAAATGACATTAAGGAAAGTTCTGCTGTTTTTAGCAAAAGCGTCCAGAAAGGAGAGCTTAGCAGGAGTCCTAGCCCTTTCACCCATACACATTTGGCTCAGGGTTACCGAAGAGGGGCCAAGAAATTAGAGTCCTCAGAAGAGAACTTATCTAGTGAGGATGAAGAGCTTCCCTGCTTCCAACACTTGTTATTTGGTAAAGTAAACAATATACCTTCTCAGTCTACTAGGCATAGCACCGTTGCTACCGAGTGTCTGTCTAAGAACACAGAGGAGAATTTATTATCATTGAAGAATAGCTTAAATGACTGCAGTAACCAGGTAATATTGGCAAAGGCATCTCAGGAACATCACCTTAGTGAGGAAACAAAATGTTCTGCTAGCTTGTTTTCTTCACAGTGCAGTGAATTGGAAGACTTGACTGCAAATACAAACACCCAGGATCCTTTCTTGATTGGTTCTTCCAAACAAATGAGGCATCAGTCTGAAAGCCAGGGAGTTGGTCTGAGTGACAAGGAATTGGTTTCAGATGATGAAGAAAGAGGAACGGGCTTGGAAGAAAATAATCAAGAAGAGCAAAGCATGGATTCAAACTTAGGTGAAGCAGCATCTGGGTGTGAGAGTGAAACAAGCGTCTCTGAAGACTGCTCAGGGCTATCCTCTCAGAGTGACATTTTAACCACTCAGCAGAGGGATACCATGCAACATAACCTGATAAAGCTCCAGCAGGAAATGGCTGAACTAGAAGCTGTGTTAGAACAGCATGGGAGCCAGCCTTCTAACAGCTACCCTTCCATCATAAGTGACTCTTCTGCCCTTGAGGACCTGCGAAATCCAGAACAAAGCACATCAGAAAAA***GGC***AGTATTAACTTCACAGAAAAGTAGTGAATACCCTATAAGCCAGAATCCAGAAGGCCTTTCTGCTGACAAGTTTGAGGTGTCTGCAGATAGTTCTACCAGTAAAAATAAAGAACCAGGAGTGGAAAGGTCATCCCCTTCTAAATGCCCATCATTAGATGATAGGTGGTACATGCACAGTTGCTCTGGGAGTCTTCAGAATAGAAACTACCCATCTCAAGAGGAGCTCATTAAGGTTGTTGATGTGGAGGAGCAACAGCTGGAAGAGTCTGGGCCACACGATTTGACGGAAACATCTTACTTGCCAAGGCAAGATCTAGAGGGAACCCCTTACCTGGAATCTGGAATCAGCCTCTTCTCTGATGACCCTGAATCTGATCCTTCTGAAGACAGAGCCCCAGAGTCAGCTCGTGTTGGCAACATACCATCTTCAACCTCTGCATTGAAAGTTCCCCAATTGAAAGTTGCAGAATCTGCCCAGAGTCCAGCTGCTGCTCATACTACTGATACTGCTGGGTATAATGCAATGGAAGAAAGTGTGAGCAGGGAGAAGCCAGAATTGACAGCTTCAACAGAAAGGGTCAACAAAAGAATGTCCATGGTGGTGTCTGGCCTGACCCCAGAAGAATTTATGCTCGTGTACAAGTTTGCCAGAAAACACCACATCACTTTAACTAATCTAATTACTGAAGAGACTACTCATGTTGTTATGAAAACAGATGCTGAGTTTGTGTGTGAACGGACACTGAAATATTTTCTAGGAATTGCGGGAGGAAAATGGGTAGTTAGCTATTTCTGGGTGACCCAGTCTATTAAAGAAAGAAAAATGCTGAATGAGCATGATTTTGAAGTCAGAGGAGATGTGGTCAATGGAAGAAACCACCAAGGTCCAAAGCGAGCAAGAGAATCCCAGGACAGAAAGATCTTCAGGGGGCTAGAAATCTGTTGCTATGGGCCCTTCACCAACATGCCCACAGATCAACTGGAATGGATGGTACAGCTGTGTGGTGCTTCTGTGGTGAAGGAGCTTTCATCATTCACCCTTGGCACAGGTGTCCACCCAATTGTGGTTGTGCAGCCAGATGCCTGGACAGAGGACAATGGCTTCCATGCAATTGGGCAGATGTGTGAGGCACCTGTGGTGACCCGAGAGTGGGTGTTGGACAGTGTAGCACTCTACCAGTGCCAGGAGCTGGACACCTACCTGATACCCCAGATCCCCCACAGCCACTACTGA

**>BRCA1 WT protein (1863AA)**

MDLSALRVEEVQNVINAMQKILECPICLELIKEPVSTKCDHIFCKFCMLKLLNQKKGPSQCPLCKNDITKRSLQESTRFSQLVEELLKIICAFQLDTGLEYANSYNFAKKENNSPEHLKDEVSIIQSMGYRNRAKRLLQSEPENPSLQETSLSVQLSNLGTVRTLRTKQRIQPQKTSVYIELGSDSSEDTVNKATYCSVGDQELLQITPQGTRDEISLDSAKKAACEFSETDVTNTEHHQPSNNDLNTTEKRAAERHPEKYQGSSVSNLHVEPCGTNTHASSLQHENSSLLLTKDRMNVEKAEFCNKSKQPGLARSQHNRWAGSKETCNDRRTPSTEKKVDLNADPLCERKEWNKQKLPCSENPRDTEDVPWITLNSSIQKVNEWFSRSDELLGSDDSHDGESESNAKVADVLDVLNEVDEYSGSSEKIDLLASDPHEALICKSERVHSKSVESNIEDKIFGKTYRKKASLPNLSHVTENLIIGAFVTEPQIIQERPLTNKLKRKRRPTSGLHPEDFIKKADLAVQKTPEMINQGTNQTEQNGQVMNITNSGHENKTKGD
SIQNEKNPNPIESLEKESAFKTKAEPISSSISNMELELNIHNSKAPKKNRLRRKSSTRHIHALELVVSRNLSPPNCTELQIDSCSSSEEIKKKKYNQMPVRHSRNLQLMEGKEPATGAKKSNKPNEQTSKRHDSDTFPELKLTNAPGSFTKCSNTSELKEFVNPSLPREEKEEKLETVKVSNNAEDPKDLMLSGERVLQTERSVESSSISLVPGTDYGTQESISLLEVSTLGKAKTEPNKCVSQCAAFENPKGLIHGCSKDNRNDTEGFKYPLGHEVNHSRETSIEMEESELDAQYLQNTFKVSKRQSFAPFSNPGNAEEECATFSAHSGSLKKQSPKVTFECEQKEENQGKNESNIKPVQTVNITAGFPVVGQKDKPVDNAKCSIKGGSRFCLSSQFRGNETGLITPNKHGLLQNPYRIPPLFPIKSFVKTKCKKNLLEENFEEHSMSPEREMGNENIPSTVSTISRNNIRENVFKEASSSNINEVGSSTNEVGSSINEIGSSDENIQAELGRNRGPKLNAMLRLGVLQPEVYKQSLPGSNCKHPEIKKQEYEEVVQTV
NTDFSPYLISDNLEQPMGSSHASQVCSETPDDLLDDGEIKEDTSFAENDIKESSAVFSKSVQKGELSRSPSPFTHTHLAQGYRRGAKKLESSEENLSSEDEELPCFQHLLFGKVNNIPSQSTRHSTVATECLSKNTEENLLSLKNSLNDCSNQVILAKASQEHHLSEETKCSASLFSSQCSELEDLTANTNTQDPFLIGSSKQMRHQSESQGVGLSDKELVSDDEERGTGLEENNQEEQSMDSNLGEAASGCESETSVSEDCSGLSSQSDILTTQQRDTMQHNLIKLQQEMAELEAVLEQHGSQPSNSYPSIISDSSALEDLRNPEQSTSEKAVLTSQKSSEYPISQNPEGLSADKFEVSADSSTSKNKEPGVERSSPSKCPSLDDRWYMHSCSGSLQNRNYPSQEELIKVVDVEEQQLEESGPHDLTETSYLPRQDLEGTPYLESGISLFSDDPESDPSEDRAPESARVGNIPSSTSALKVPQLKVAESAQSPAAAHTTDTAGYNAMEESVSREKPELTASTERVNKRMSMVVSGLTPEEFMLVYKFARKHHITLTNLI
TEETTHVVMKTDAEFVCERTLKYFLGIAGGKWVVSYFWVTQSIKERKMLNEHDFEVRGDVVNGRNHQGPKRARESQDRKIFRGLEICCYGPFTNMPTDQLEWMVQLCGASVVKELSSFTLGTGVHPIVVVQPDAWTEDNGFHAIGQMCEAPVVTREWVLDSVALYQCQELDTYLIPQIPHSHY

**>BRCA1 c.4358-2A>G protein (1863AA)**

MDLSALRVEEVQNVINAMQKILECPICLELIKEPVSTKCDHIFCKFCMLKLLNQKKGPSQCPLCKNDITKRSLQESTRFSQLVEELLKIICAFQLDTGLEYANSYNFAKKENNSPEHLKDEVSIIQSMGYRNRAKRLLQSEPENPSLQETSLSVQLSNLGTVRTLRTKQRIQPQKTSVYIELGSDSSEDTVNKATYCSVGDQELLQITPQGTRDEISLDSAKKAACEFSETDVTNTEHHQPSNNDLNTTEKRAAERHPEKYQGSSVSNLHVEPCGTNTHASSLQHENSSLLLTKDRMNVEKAEFCNKSKQPGLARSQHNRWAGSKETCNDRRTPSTEKKVDLNADPLCERKEWNKQKLPCSENPRDTEDVPWITLNSSIQKVNEWFSRSDELLGSDDSHDGESESNAKVADVLDVLNEVDEYSGSSEKIDLLASDPHEALICKSERVHSKSVESNIEDKIFGKTYRKKASLPNLSHVTENLIIGAFVTEPQIIQERPLTNKLKRKRRPTSGLHPEDFIKKADLAVQKTPEMINQGTNQTEQNGQVMNITNSGHENKTKGDSIQNEKNPNPIESLEKESAFKTKAEPISSSISNMELELNIHNSKAPKKNRLRRKSSTRHIHALELVVSRNLSPPNCTELQIDSCSSSEEIKKKKYNQMPVRHSRNLQLMEGKEPATGAKKSNKPNEQTSKRHDSDTFPELKLTNAPGSFTKCSNTSELKEFVNPSLPREEKEEKLETVKVSNNAEDPKDLMLSGERVLQTERSVESSSISLVPGTDYGTQESISLLEVSTLGKAKTEPNKCVSQCAAFENPKGLIHGCSKDNRNDTEGFKYPLGHEVNHSRETSIEMEESELDAQYLQNTFKVSKRQSFAPFSNPGNAEEECATFSAHSGSLKKQSPKVTFECEQKEENQGKNESNIKPVQTVNITAGFPVVGQKDKPVDNAKCSIKGGSRFCLSSQFRGNETGLITPNKHGLLQNPYRIPPLFPIKSFVKTKCKKNLLEENFEEHSMSPEREMGNENIPSTVSTISRNNIRENVFKEASSSNINEVGSSTNEVGSSINEIGSSDENIQAELGRNRGPKLNAMLRLGVLQPEVYKQSLPGSNCKHPEIKKQEYEEVVQTVNTDFSPYLISDNLEQPMGSSHASQVCSETPDDLLDDGEIKEDTSFAENDIKESSAVFSKSVQKGELSRSPSPFTHTHLAQGYRRGAKKLESSEENLSSEDEELPCFQHLLFGKVNNIPSQSTRHSTVATECLSKNTEENLLSLKNSLNDCSNQVILAKASQEHHLSEETKCSASLFSSQCSELEDLTANTNTQDPFLIGSSKQMRHQSESQGVGLSDKELVSDDEERGTGLEENNQEEQSMDSNLGEAASGCESETSVSEDCSGLSSQSDILTTQQRDTMQHNLIKLQQEMAELEAVLEQHGSQPSNSYPSIISDSSALEDLRNPEQSTSEKGSINFTEK--IPYKPESRRPFC-QV-GVCR-FYQ-K-RTRSGKVIPF-MPIIR--VVHAQLLWESSE-KLPISRGAH-GC-CGGATAGRVWATRFDGNILLAKARSRGNPLPGIWNQPLL--P-I-SF-RQSPRVSSCWQHTIFNLCIESSPIESCRICPESSCCSYY-YCWV-CNGRKCEQGEARIDSFNRKGQQKNVHGGVWPDPRRIYARVQVCQKTPHHFN-SNY-RDYSCCYENRC-VCV-TDTEIFSRNCGRKMGS-LFLGDPVY-RKKNAE-A-F-SQRRCGQWKKPPRSKASKRIPGQKDLQGARNLLLWALHQHAHRSTGMDGTAVWCFCGEGAFIIHPWHRCPPNCGCAARCLDRGQWLPCNWADV-GTCGDPRVGVGQCSTLPVPGAGHLPDTPDPPQPLL

**p.(Ala1453Glyfs)**

**BRCA2 [Homo sapiens(human)]**

Fullname: BRCA2

TranscriptID: NM_000059.4

Mutation: c.475+5G>C

Chromosome: 13

**>BRCA2 WT Nucleotide Sequence (10257nt):**ATGCCTATTGGATCCAAAGAGAGGCCAACATTTTTTGAAATTTTTAAGACACGCTGCAACAAAGCAGATTTAGGACCAATAAGTCTTAATTGGTTTGAAGAACTTTCTTCAGAAGCTCCACCCTATAATTCTGAACCTGCAGAAGAATCTGAACATAAAAACAACAATTACGAACCAAACCTATTTAAAACTCCACAAAGGAAACCATCTTATAATCAGCTGGCTTCAACTCCAATAATATTCAAAGAGCAAGGGCTGACTCTGCCGCTGTACCAATCTCCTGTAAAAGAATTAGATAAATTCAAATTAGACTTAGGAAGGAATGTTCCCAATAGTAGACATAAAAGTCTTCGCACAGTGAAAACTAAAATGGATCAAGCAGATGATGTTTCCTGTCCACTTCTAAATTCTTGTCTTAGTGAAAG***TCCTGTTGTTCTACAATGTACACATGTAACACCACAAAGAGATAAGTCAG***TGGTATGTGGGAGTTTGTTTCATACACCAAAGTTTGTGAAGGGTCGTCAGACACCAAAACATATTTCTGAAAGTCTAGGAGCTGAGGTGGATCCTGATATGTCTTGGTCAAGTTCTTTAGCTACACCACCCACCCTTAGTTCTACTGTGCTCATAGTCAGAAATGAAGAAGCATCTGAAACTGTATTTCCTCATGATACTACTGCTAATGTGAAAAGCTATTTTTCCAATCATGATGAAAGTCTGAAGAAAAATGATAGATTTATCGCTTCTGTGACAGACAGTGAAAACACAAATCAAAGAGAAGCTGCAAGTCATGGATTTGGAAAAACATCAGGGAATTCATTTAAAGTAAATAGCTGCAAAGACCACATTGGAAAGTCAATGCCAAATGTCCTAGAAGATGAAGTATATGAAACAGTTGTAGATACCTCTGAAGAAGATAGTTTTTCATTATGTTTTTCTAAATGTAGAACAAAAAATCTACAAAAAGTAAGAACTAGCAAGACTAGGAAAAAAATTTTCCATGAAGCAAACGCTGATGAATGTGAAAAATCTAAAAACCAAGTGAAAGAAAAATACTCATTTGTATCTGAAGTGGAACCAAATGATACTGATCCATTAGATTCAAATGTAGCAAATCAGAAGCCCTTTGAGAGTGGAAGTGACAAAATCTCCAAGGAAGTTGTACCGTCTTTGGCCTGTGAATGGTCTCAACTAACCCTTTCAGGTCTAAATGGAGCCCAGATGGAGAAAATACCCCTATTGCATATTTCTTCATGTGACCAAAATATTTCAGAAAAAGACCTATTAGACACAGAGAACAAAAGAAAGAAAGATTTTCTTACTTCAGAGAATTCTTTGCCACGTATTTCTAGCCTACCAAAATCAGAGAAGCCATTAAATGAGGAAACAGTGGTAAATAAGAGAGATGAAGAGCAGCATCTTGAATCTCATACAGACTGCATTCTTGCAGTAAAGCAGGCAATATCTGGAACTTCTCCAGTGGCTTCTTCATTTCAGGGTATCAAAAAGTCTATATTCAGAATAAGAGAATCACCTAAAGAGACTTTCAATGCAAGTTTTTCAGGTCATATGACTGATCCAAACTTTAAAAAAGAAACTGAAGCCTCTGAAAGTGGACTGGAAATACATACTGTTTGCTCACAGAAGGAGGACTCCTTATGTCCAAATTTAATTGATAATGGAAGCTGGCCAGCCACCACCACACAGAATTCTGTAGCTTTGAAGAATGCAGGTTTAATATCCACTTTGAAAAAGAAAACAAATAAGTTTATTTATGCTATACATGATGAAACATCTTATAAAGGAAAAAAAATACCGAAAGACCAAAAATCAGAACTAATTAACTGTTCAGCCCAGTTTGAAGCAAATGCTTTTGAAGCACCACTTACATTTGCAAATGCTGATTCAGGTTTATTGCATTCTTCTGTGAAAAGAAGCTGTTCACAGAATGATTCTGAAGAACCAACTTTGTCCTTAACTAGCTCTTTTGGGACAATTCTGAGGAAATGTTCTAGAAATGAAACATGTTCTAATAATACAGTAATCTCTCAGGATCTTGATTATAAAGAAGCAAAATGTAATAAGGAAAAACTACAGTTATTTATTACCCCAGAAGCTGATTCTCTGTCATGCCTGCAGGAAGGACAGTGTGAAAATGATCCAAAAAGCAAAAAAGTTTCAGATATAAAAGAAGAGGTCTTGGCTGCAGCATGTCACCCAGTACAACATTCAAAAGTGGAATACAGTGATACTGACTTTCAATCCCAGAAAAGTCTTTTATATGATCATGAAAATGCCAGCACTCTTATTTTAACTCCTACTTCCAAGGATGTTCTGTCAAACCTAGTCATGATTTCTAGAGGCAAAGAATCATACAAAATGTCAGACAAGCTCAAAGGTAACAATTATGAATCTGATGTTGAATTAACCAAAAATATTCCCATGGAAAAGAATCAAGATGTATGTGCTTTAAATGAAAATTATAAAAACGTTGAGCTGTTGCCACCTGAAAAATACATGAGAGTAGCATCACCTTCAAGAAAGGTACAATTCAACCAAAACACAAATCTAAGAGTAATCCAAAAAAATCAAGAAGAAACTACTTCAATTTCAAAAATAACTGTCAATCCAGACTCTGAAGAACTTTTCTCAGACAATGAGAATAATTTTGTCTTCCAAGTAGCTAATGAAAGGAATAATCTTGCTTTAGGAAATACTAAGGAACTTCATGAAACAGACTTGACTTGTGTAAACGAACCCATTTTCAAGAACTCTACCATGGTTTTATATGGAGACACAGGTGATAAACAAGCAACCCAAGTGTCAATTAAAAAAGATTTGGTTTATGTTCTTGCAGAGGAGAACAAAAATAGTGTAAAGCAGCATATAAAAATGACTCTAGGTCAAGATTTAAAATCGGACATCTCCTTGAATATAGATAAAATACCAGAAAAAAATAATGATTACATGAACAAATGGGCAGGACTCTTAGGTCCAATTTCAAATCACAGTTTTGGAGGTAGCTTCAGAACAGCTTCAAATAAGGAAATCAAGCTCTCTGAACATAACATTAAGAAGAGCAAAATGTTCTTCAAAGATATTGAAGAACAATATCCTACTAGTTTAGCTTGTGTTGAAATTGTAAATACCTTGGCATTAGATAATCAAAAGAAACTGAGCAAGCCTCAGTCAATTAATACTGTATCTGCACATTTACAGAGTAGTGTAGTTGTTTCTGATTGTAAAAATAGTCATATAACCCCTCAGATGTTATTTTCCAAGCAGGATTTTAATTCAAACCATAATTTAACACCTAGCCAAAAGGCAGAAATTACAGAACTTTCTACTATATTAGAAGAATCAGGAAGTCAGTTTGAATTTACTCAGTTTAGAAAACCAAGCTACATATTGCAGAAGAGTACATTTGAAGTGCCTGAAAACCAGATGACTATCTTAAAGACCACTTCTGAGGAATGCAGAGATGCTGATCTTCATGTCATAATGAATGCCCCATCGATTGGTCAGGTAGACAGCAGCAAGCAATTTGAAGGTACAGTTGAAATTAAACGGAAGTTTGCTGGCCTGTTGAAAAATGACTGTAACAAAAGTGCTTCTGGTTATTTAACAGATGAAAATGAAGTGGGGTTTAGGGGCTTTTATTCTGCTCATGGCACAAAACTGAATGTTTCTACTGAAGCTCTGCAAAAAGCTGTGAAACTGTTTAGTGATATTGAGAATATTAGTGAGGAAACTTCTGCAGAGGTACATCCAATAAGTTTATCTTCAAGTAAATGTCATGATTCTGTTGTTTCAATGTTTAAGATAGAAAATCATAATGATAAAACTGTAAGTGAAAAAAATAATAAATGCCAACTGATATTACAAAATAATATTGAAATGACTACTGGCACTTTTGTTGAAGAAATTACTGAAAATTACAAGAGAAATACTGAAAATGAAGATAACAAATATACTGCTGCCAGTAGAAATTCTCATAACTTAGAATTTGATGGCAGTGATTCAAGTAAAAATGATACTGTTTGTATTCATAAAGATGAAACGGACTTGCTATTTACTGATCAGCACAACATATGTCTTAAATTATCTGGCCAGTTTATGAAGGAGGGAAACACTCAGATTAAAGAAGATTTGTCAGATTTAACTTTTTTGGAAGTTGCGAAAGCTCAAGAAGCATGTCATGGTAATACTTCAAATAAAGAACAGTTAACTGCTACTAAAACGGAGCAAAATATAAAAGATTTTGAGACTTCTGATACATTTTTTCAGACTGCAAGTGGGAAAAATATTAGTGTCGCCAAAGAGTCATTTAATAAAATTGTAAATTTCTTTGATCAGAAACCAGAAGAATTGCATAACTTTTCCTTAAATTCTGAATTACATTCTGACATAAGAAAGAACAAAATGGACATTCTAAGTTATGAGGAAACAGACATAGTTAAACACAAAATACTGAAAGAAAGTGTCCCAGTTGGTACTGGAAATCAACTAGTGACCTTCCAGGGACAACCCGAACGTGATGAAAAGATCAAAGAACCTACTCTATTGGGTTTTCATACAGCTAGCGGGAAAAAAGTTAAAATTGCAAAGGAATCTTTGGACAAAGTGAAAAACCTTTTTGATGAAAAAGAGCAAGGTACTAGTGAAATCACCAGTTTTAGCCATCAATGGGCAAAGACCCTAAAGTACAGAGAGGCCTGTAAAGACCTTGAATTAGCATGTGAGACCATTGAGATCACAGCTGCCCCAAAGTGTAAAGAAATGCAGAATTCTCTCAATAATGATAAAAACCTTGTTTCTATTGAGACTGTGGTGCCACCTAAGCTCTTAAGTGATAATTTATGTAGACAAACTGAAAATCTCAAAACATCAAAAAGTATCTTTTTGAAAGTTAAAGTACATGAAAATGTAGAAAAAGAAACAGCAAAAAGTCCTGCAACTTGTTACACAAATCAGTCCCCTTATTCAGTCATTGAAAATTCAGCCTTAGCTTTTTACACAAGTTGTAGTAGAAAAACTTCTGTGAGTCAGACTTCATTACTTGAAGCAAAAAAATGGCTTAGAGAAGGAATATTTGATGGTCAACCAGAAAGAATAAATACTGCAGATTATGTAGGAAATTATTTGTATGAAAATAATTCAAACAGTACTATAGCTGAAAATGACAAAAATCATCTCTCCGAAAAACAAGATACTTATTTAAGTAACAGTAGCATGTCTAACAGCTATTCCTACCATTCTGATGAGGTATATAATGATTCAGGATATCTCTCAAAAAATAAACTTGATTCTGGTATTGAGCCAGTATTGAAGAATGTTGAAGATCAAAAAAACACTAGTTTTTCCAAAGTAATATCCAATGTAAAAGATGCAAATGCATACCCACAAACTGTAAATGAAGATATTTGCGTTGAGGAACTTGTGACTAGCTCTTCACCCTGCAAAAATAAAAATGCAGCCATTAAATTGTCCATATCTAATAGTAATAATTTTGAGGTAGGGCCACCTGCATTTAGGATAGCCAGTGGTAAAATCGTTTGTGTTTCACATGAAACAATTAAAAAAGTGAAAGACATATTTACAGACAGTTTCAGTAAAGTAATTAAGGAAAACAACGAGAATAAATCAAAAATTTGCCAAACGAAAATTATGGCAGGTTGTTACGAGGCATTGGATGATTCAGAGGATATTCTTCATAACTCTCTAGATAATGATGAATGTAGCACGCATTCACATAAGGTTTTTGCTGACATTCAGAGTGAAGAAATTTTACAACATAACCAAAATATGTCTGGATTGGAGAAAGTTTCTAAAATATCACCTTGTGATGTTAGTTTGGAAACTTCAGATATATGTAAATGTAGTATAGGGAAGCTTCATAAGTCAGTCTCATCTGCAAATACTTGTGGGATTTTTAGCACAGCAAGTGGAAAATCTGTCCAGGTATCAGATGCTTCATTACAAAACGCAAGACAAGTGTTTTCTGAAATAGAAGATAGTACCAAGCAAGTCTTTTCCAAAGTATTGTTTAAAAGTAACGAACATTCAGACCAGCTCACAAGAGAAGAAAATACTGCTATACGTACTCCAGAACATTTAATATCCCAAAAAGGCTTTTCATATAATGTGGTAAATTCATCTGCTTTCTCTGGATTTAGTACAGCAAGTGGAAAGCAAGTTTCCATTTTAGAAAGTTCCTTACACAAAGTTAAGGGAGTGTTAGAGGAATTTGATTTAATCAGAACTGAGCATAGTCTTCACTATTCACCTACGTCTAGACAAAATGTATCAAAAATACTTCCTCGTGTTGATAAGAGAAACCCAGAGCACTGTGTAAACTCAGAAATGGAAAAAACCTGCAGTAAAGAATTTAAATTATCAAATAACTTAAATGTTGAAGGTGGTTCTTCAGAAAATAATCACTCTATTAAAGTTTCTCCATATCTCTCTCAATTTCAACAAGACAAACAACAGTTGGTATTAGGAACCAAAGTGTCACTTGTTGAGAACATTCATGTTTTGGGAAAAGAACAGGCTTCACCTAAAAACGTAAAAATGGAAATTGGTAAAACTGAAACTTTTTCTGATGTTCCTGTGAAAACAAATATAGAAGTTTGTTCTACTTACTCCAAAGATTCAGAAAACTACTTTGAAACAGAAGCAGTAGAAATTGCTAAAGCTTTTATGGAAGATGATGAACTGACAGATTCTAAACTGCCAAGTCATGCCACACATTCTCTTTTTACATGTCCCGAAAATGAGGAAATGGTTTTGTCAAATTCAAGAATTGGAAAAAGAAGAGGAGAGCCCCTTATCTTAGTGGGAGAACCCTCAATCAAAAGAAACTTATTAAATGAATTTGACAGGATAATAGAAAATCAAGAAAAATCCTTAAAGGCTTCAAAAAGCACTCCAGATGGCACAATAAAAGATCGAAGATTGTTTATGCATCATGTTTCTTTAGAGCCGATTACCTGTGTACCCTTTCGCACAACTAAGGAACGTCAAGAGATACAGAATCCAAATTTTACCGCACCTGGTCAAGAATTTCTGTCTAAATCTCATTTGTATGAACATCTGACTTTGGAAAAATCTTCAAGCAATTTAGCAGTTTCAGGACATCCATTTTATCAAGTTTCTGCTACAAGAAATGAAAAAATGAGACACTTGATTACTACAGGCAGACCAACCAAAGTCTTTGTTCCACCTTTTAAAACTAAATCACATTTTCACAGAGTTGAACAGTGTGTTAGGAATATTAACTTGGAGGAAAACAGACAAAAGCAAAACATTGATGGACATGGCTCTGATGATAGTAAAAATAAGATTAATGACAATGAGATTCATCAGTTTAACAAAAACAACTCCAATCAAGCAGTAGCTGTAACTTTCACAAAGTGTGAAGAAGAACCTTTAGATTTAATTACAAGTCTTCAGAATGCCAGAGATATACAGGATATGCGAATTAAGAAGAAACAAAGGCAACGCGTCTTTCCACAGCCAGGCAGTCTGTATCTTGCAAAAACATCCACTCTGCCTCGAATCTCTCTGAAAGCAGCAGTAGGAGGCCAAGTTCCCTCTGCGTGTTCTCATAAACAGCTGTATACGTATGGCGTTTCTAAACATTGCATAAAAATTAACAGCAAAAATGCAGAGTCTTTTCAGTTTCACACTGAAGATTATTTTGGTAAGGAAAGTTTATGGACTGGAAAAGGAATACAGTTGGCTGATGGTGGATGGCTCATACCCTCCAATGATGGAAAGGCTGGAAAAGAAGAATTTTATAGGGCTCTGTGTGACACTCCAGGTGTGGATCCAAAGCTTATTTCTAGAATTTGGGTTTATAATCACTATAGATGGATCATATGGAAACTGGCAGCTATGGAATGTGCCTTTCCTAAGGAATTTGCTAATAGATGCCTAAGCCCAGAAAGGGTGCTTCTTCAACTAAAATACAGATATGATACGGAAATTGATAGAAGCAGAAGATCGGCTATAAAAAAGATAATGGAAAGGGATGACACAGCTGCAAAAACACTTGTTCTCTGTGTTTCTGACATAATTTCATTGAGCGCAAATATATCTGAAACTTCTAGCAATAAAACTAGTAGTGCAGATACCCAAAAAGTGGCCATTATTGAACTTACAGATGGGTGGTATGCTGTTAAGGCCCAGTTAGATCCTCCCCTCTTAGCTGTCTTAAAGAATGGCAGACTGACAGTTGGTCAGAAGATTATTCTTCATGGAGCAGAACTGGTGGGCTCTCCTGATGCCTGTACACCTCTTGAAGCCCCAGAATCTCTTATGTTAAAGATTTCTGCTAACAGTACTCGGCCTGCTCGCTGGTATACCAAACTTGGATTCTTTCCTGACCCTAGACCTTTTCCTCTGCCCTTATCATCGCTTTTCAGTGATGGAGGAAATGTTGGTTGTGTTGATGTAATTATTCAAAGAGCATACCCTATACAGTGGATGGAGAAGACATCATCTGGATTATACATATTTCGCAATGAAAGAGAGGAAGAAAAGGAAGCAGCAAAATATGTGGAGGCCCAACAAAAGAGACTAGAAGCCTTATTCACTAAAATTCAGGAGGAATTTGAAGAACATGAAGAAAACACAACAAAACCATATTTACCATCACGTGCACTAACAAGACAGCAAGTTCGTGCTTTGCAAGATGGTGCAGAGCTTTATGAAGCAGTGAAGAATGCAGCAGACCCAGCTTACCTTGAGGGTTATTTCAGTGAAGAGCAGTTAAGAGCCTTGAATAATCACAGGCAAATGTTGAATGATAAGAAACAAGCTCAGATCCAGTTGGAAATTAGGAAGGCCATGGAATCTGCTGAACAAAAGGAACAAGGTTTATCAAGGGATGTCACAACCGTGTGGAAGTTGCGTATTGTAAGCTATTCAAAAAAAGAAAAAGATTCAGTTATACTGAGTATTTGGCGTCCATCATCAGATTTATATTCTCTGTTAACAGAAGGAAAGAGATACAGAATTTATCATCTTGCAACTTCAAAATCTAAAAGTAAATCTGAAAGAGCTAACATACAGTTAGCAGCGACAAAAAAAACTCAGTATCAACAACTACCGGTTTCAGATGAAATTTTATTTCAGATTTACCAGCCACGGGAGCCCCTTCACTTCAGCAAATTTTTAGATCCAGACTTTCAGCCATCTTGTTCTGAGGTGGACCTAATAGGATTTGTCGTTTCTGTTGTGAAAAAAACAGGACTTGCCCCTTTCGTCTATTTGTCAGACGAATGTTACAATTTACTGGCAATAAAGTTTTGGATAGACCTTAATGAGGACATTATTAAGCCTCATATGTTAATTGCTGCAAGCAACCTCCAGTGGCGACCAGAATCCAAATCAGGCCTTCTTACTTTATTTGCTGGAGATTTTTCTGTGTTTTCTGCTAGTCCAAAAGAGGGCCACTTTCAAGAGACATTCAACAAAATGAAAAATACTGTTGAGAATATTGACATACTTTGCAATGAAGCAGAAAACAAGCTTATGCATATACTGCATGCAAATGATCCCAAGTGGTCCACCCCAACTAAAGACTGTACTTCAGGGCCGTACACTGCTCAAATCATTCCTGGTACAGGAAACAAGCTTCTGATGTCTTCTCCTAATTGTGAGATATATTATCAAAGTCCTTTATCACTTTGTATGGCCAAAAGGAAGTCTGTTTCCACACCTGTCTCAGCCCAGATGACTTCAAAGTCTTGTAAAGGGGAGAAAGAGATTGATGACCAAAAGAACTGCAAAAAGAGAAGAGCCTTGGATTTCTTGAGTAGACTGCCTTTACCTCCACCTGTTAGTCCCATTTGTACATTTGTTTCTCCGGCTGCACAGAAGGCATTTCAGCCACCAAGGAGTTGTGGCACCAAATACGAAACACCCATAAAGAAAAAAGAACTGAATTCTCCTCAGATGACTCCATTTAAAAAATTCAATGAAATTTCTCTTTTGGAAAGTAATTCAATAGCTGACGAAGAACTTGCATTGATAAATACCCAAGCTCTTTTGTCTGGTTCAACAGGAGAAAAACAATTTATATCTGTCAGTGAATCCACTAGGACTGCTCCCACCAGTTCAGAAGATTATCTCAGACTGAAACGACGTTGTACTACATCTCTGATCAAAGAACAGGAGAGTTCCCAGGCCAGTACGGAAGAATGTGAGAAAAATAAGCAGGACACAATTACAACTAAAAAATATATCTAA

**>BRCA2 c.475+5G>C Nucleotide Sequence (10257nt):**ATGCCTATTGGATCCAAAGAGAGGCCAACATTTTTTGAAATTTTTAAGACACGCTGCAACAAAGCAGATTTAGGACCAATAAGTCTTAATTGGTTTGAAGAACTTTCTTCAGAAGCTCCACCCTATAATTCTGAACCTGCAGAAGAATCTGAACATAAAAACAACAATTACGAACCAAACCTATTTAAAACTCCACAAAGGAAACCATCTTATAATCAGCTGGCTTCAACTCCAATAATATTCAAAGAGCAAGGGCTGACTCTGCCGCTGTACCAATCTCCTGTAAAAGAATTAGATAAATTCAAATTAGACTTAGGAAGGAATGTTCCCAATAGTAGACATAAAAGTCTTCGCACAGTGAAAACTAAAATGGATCAAGCAGATGATGTTTCCTGTCCACTTCTAAATTCTTGTCTTAGTGAAAGT**GGT**ATGTGGGAGTTTGTTTCATACACCAAAGTTTGTGAAGGGTCGTCAGACACCAAAACATATTTC**TGA**AAGTCTAGGAGCTGAGGTGGATCCTGATATGTCTTGGTCAAGTTCTTTAGCTACACCACCCACCCTTAGTTCTACTGTGCTCATAGTCAGAAATGAAGAAGCATCTGAAACTGTATTTCCTCATGATACTACTGCTAATGTGAAAAGCTATTTTTCCAATCATGATGAAAGTCTGAAGAAAAATGATAGATTTATCGCTTCTGTGACAGACAGTGAAAACACAAATCAAAGAGAAGCTGCAAGTCATGGATTTGGAAAAACATCAGGGAATTCATTTAAAGTAAATAGCTGCAAAGACCACATTGGAAAGTCAATGCCAAATGTCCTAGAAGATGAAGTATATGAAACAGTTGTAGATACCTCTGAAGAAGATAGTTTTTCATTATGTTTTTCTAAATGTAGAACAAAAAATCTACAAAAAGTAAGAACTAGCAAGACTAGGAAAAAAATTTTCCATGAAGCAAACGCTGATGAATGTGAAAAATCTAAAAACCAAGTGAAAGAAAAATACTCATTTGTATCTGAAGTGGAACCAAATGATACTGATCCATTAGATTCAAATGTAGCAAATCAGAAGCCCTTTGAGAGTGGAAGTGACAAAATCTCCAAGGAAGTTGTACCGTCTTTGGCCTGTGAATGGTCTCAACTAACCCTTTCAGGTCTAAATGGAGCCCAGATGGAGAAAATACCCCTATTGCATATTTCTTCATGTGACCAAAATATTTCAGAAAAAGACCTATTAGACACAGAGAACAAAAGAAAGAAAGATTTTCTTACTTCAGAGAATTCTTTGCCACGTATTTCTAGCCTACCAAAATCAGAGAAGCCATTAAATGAGGAAACAGTGGTAAATAAGAGAGATGAAGAGCAGCATCTTGAATCTCATACAGACTGCATTCTTGCAGTAAAGCAGGCAATATCTGGAACTTCTCCAGTGGCTTCTTCATTTCAGGGTATCAAAAAGTCTATATTCAGAATAAGAGAATCACCTAAAGAGACTTTCAATGCAAGTTTTTCAGGTCATATGACTGATCCAAACTTTAAAAAAGAAACTGAAGCCTCTGAAAGTGGACTGGAAATACATACTGTTTGCTCACAGAAGGAGGACTCCTTATGTCCAAATTTAATTGATAATGGAAGCTGGCCAGCCACCACCACACAGAATTCTGTAGCTTTGAAGAATGCAGGTTTAATATCCACTTTGAAAAAGAAAACAAATAAGTTTATTTATGCTATACATGATGAAACATCTTATAAAGGAAAAAAAATACCGAAAGACCAAAAATCAGAACTAATTAACTGTTCAGCCCAGTTTGAAGCAAATGCTTTTGAAGCACCACTTACATTTGCAAATGCTGATTCAGGTTTATTGCATTCTTCTGTGAAAAGAAGCTGTTCACAGAATGATTCTGAAGAACCAACTTTGTCCTTAACTAGCTCTTTTGGGACAATTCTGAGGAAATGTTCTAGAAATGAAACATGTTCTAATAATACAGTAATCTCTCAGGATCTTGATTATAAAGAAGCAAAATGTAATAAGGAAAAACTACAGTTATTTATTACCCCAGAAGCTGATTCTCTGTCATGCCTGCAGGAAGGACAGTGTGAAAATGATCCAAAAAGCAAAAAAGTTTCAGATATAAAAGAAGAGGTCTTGGCTGCAGCATGTCACCCAGTACAACATTCAAAAGTGGAATACAGTGATACTGACTTTCAATCCCAGAAAAGTCTTTTATATGATCATGAAAATGCCAGCACTCTTATTTTAACTCCTACTTCCAAGGATGTTCTGTCAAACCTAGTCATGATTTCTAGAGGCAAAGAATCATACAAAATGTCAGACAAGCTCAAAGGTAACAATTATGAATCTGATGTTGAATTAACCAAAAATATTCCCATGGAAAAGAATCAAGATGTATGTGCTTTAAATGAAAATTATAAAAACGTTGAGCTGTTGCCACCTGAAAAATACATGAGAGTAGCATCACCTTCAAGAAAGGTACAATTCAACCAAAACACAAATCTAAGAGTAATCCAAAAAAATCAAGAAGAAACTACTTCAATTTCAAAAATAACTGTCAATCCAGACTCTGAAGAACTTTTCTCAGACAATGAGAATAATTTTGTCTTCCAAGTAGCTAATGAAAGGAATAATCTTGCTTTAGGAAATACTAAGGAACTTCATGAAACAGACTTGACTTGTGTAAACGAACCCATTTTCAAGAACTCTACCATGGTTTTATATGGAGACACAGGTGATAAACAAGCAACCCAAGTGTCAATTAAAAAAGATTTGGTTTATGTTCTTGCAGAGGAGAACAAAAATAGTGTAAAGCAGCATATAAAAATGACTCTAGGTCAAGATTTAAAATCGGACATCTCCTTGAATATAGATAAAATACCAGAAAAAAATAATGATTACATGAACAAATGGGCAGGACTCTTAGGTCCAATTTCAAATCACAGTTTTGGAGGTAGCTTCAGAACAGCTTCAAATAAGGAAATCAAGCTCTCTGAACATAACATTAAGAAGAGCAAAATGTTCTTCAAAGATATTGAAGAACAATATCCTACTAGTTTAGCTTGTGTTGAAATTGTAAATACCTTGGCATTAGATAATCAAAAGAAACTGAGCAAGCCTCAGTCAATTAATACTGTATCTGCACATTTACAGAGTAGTGTAGTTGTTTCTGATTGTAAAAATAGTCATATAACCCCTCAGATGTTATTTTCCAAGCAGGATTTTAATTCAAACCATAATTTAACACCTAGCCAAAAGGCAGAAATTACAGAACTTTCTACTATATTAGAAGAATCAGGAAGTCAGTTTGAATTTACTCAGTTTAGAAAACCAAGCTACATATTGCAGAAGAGTACATTTGAAGTGCCTGAAAACCAGATGACTATCTTAAAGACCACTTCTGAGGAATGCAGAGATGCTGATCTTCATGTCATAATGAATGCCCCATCGATTGGTCAGGTAGACAGCAGCAAGCAATTTGAAGGTACAGTTGAAATTAAACGGAAGTTTGCTGGCCTGTTGAAAAATGACTGTAACAAAAGTGCTTCTGGTTATTTAACAGATGAAAATGAAGTGGGGTTTAGGGGCTTTTATTCTGCTCATGGCACAAAACTGAATGTTTCTACTGAAGCTCTGCAAAAAGCTGTGAAACTGTTTAGTGATATTGAGAATATTAGTGAGGAAACTTCTGCAGAGGTACATCCAATAAGTTTATCTTCAAGTAAATGTCATGATTCTGTTGTTTCAATGTTTAAGATAGAAAATCATAATGATAAAACTGTAAGTGAAAAAAATAATAAATGCCAACTGATATTACAAAATAATATTGAAATGACTACTGGCACTTTTGTTGAAGAAATTACTGAAAATTACAAGAGAAATACTGAAAATGAAGATAACAAATATACTGCTGCCAGTAGAAATTCTCATAACTTAGAATTTGATGGCAGTGATTCAAGTAAAAATGATACTGTTTGTATTCATAAAGATGAAACGGACTTGCTATTTACTGATCAGCACAACATATGTCTTAAATTATCTGGCCAGTTTATGAAGGAGGGAAACACTCAGATTAAAGAAGATTTGTCAGATTTAACTTTTTTGGAAGTTGCGAAAGCTCAAGAAGCATGTCATGGTAATACTTCAAATAAAGAACAGTTAACTGCTACTAAAACGGAGCAAAATATAAAAGATTTTGAGACTTCTGATACATTTTTTCAGACTGCAAGTGGGAAAAATATTAGTGTCGCCAAAGAGTCATTTAATAAAATTGTAAATTTCTTTGATCAGAAACCAGAAGAATTGCATAACTTTTCCTTAAATTCTGAATTACATTCTGACATAAGAAAGAACAAAATGGACATTCTAAGTTATGAGGAAACAGACATAGTTAAACACAAAATACTGAAAGAAAGTGTCCCAGTTGGTACTGGAAATCAACTAGTGACCTTCCAGGGACAACCCGAACGTGATGAAAAGATCAAAGAACCTACTCTATTGGGTTTTCATACAGCTAGCGGGAAAAAAGTTAAAATTGCAAAGGAATCTTTGGACAAAGTGAAAAACCTTTTTGATGAAAAAGAGCAAGGTACTAGTGAAATCACCAGTTTTAGCCATCAATGGGCAAAGACCCTAAAGTACAGAGAGGCCTGTAAAGACCTTGAATTAGCATGTGAGACCATTGAGATCACAGCTGCCCCAAAGTGTAAAGAAATGCAGAATTCTCTCAATAATGATAAAAACCTTGTTTCTATTGAGACTGTGGTGCCACCTAAGCTCTTAAGTGATAATTTATGTAGACAAACTGAAAATCTCAAAACATCAAAAAGTATCTTTTTGAAAGTTAAAGTACATGAAAATGTAGAAAAAGAAACAGCAAAAAGTCCTGCAACTTGTTACACAAATCAGTCCCCTTATTCAGTCATTGAAAATTCAGCCTTAGCTTTTTACACAAGTTGTAGTAGAAAAACTTCTGTGAGTCAGACTTCATTACTTGAAGCAAAAAAATGGCTTAGAGAAGGAATATTTGATGGTCAACCAGAAAGAATAAATACTGCAGATTATGTAGGAAATTATTTGTATGAAAATAATTCAAACAGTACTATAGCTGAAAATGACAAAAATCATCTCTCCGAAAAACAAGATACTTATTTAAGTAACAGTAGCATGTCTAACAGCTATTCCTACCATTCTGATGAGGTATATAATGATTCAGGATATCTCTCAAAAAATAAACTTGATTCTGGTATTGAGCCAGTATTGAAGAATGTTGAAGATCAAAAAAACACTAGTTTTTCCAAAGTAATATCCAATGTAAAAGATGCAAATGCATACCCACAAACTGTAAATGAAGATATTTGCGTTGAGGAACTTGTGACTAGCTCTTCACCCTGCAAAAATAAAAATGCAGCCATTAAATTGTCCATATCTAATAGTAATAATTTTGAGGTAGGGCCACCTGCATTTAGGATAGCCAGTGGTAAAATCGTTTGTGTTTCACATGAAACAATTAAAAAAGTGAAAGACATATTTACAGACAGTTTCAGTAAAGTAATTAAGGAAAACAACGAGAATAAATCAAAAATTTGCCAAACGAAAATTATGGCAGGTTGTTACGAGGCATTGGATGATTCAGAGGATATTCTTCATAACTCTCTAGATAATGATGAATGTAGCACGCATTCACATAAGGTTTTTGCTGACATTCAGAGTGAAGAAATTTTACAACATAACCAAAATATGTCTGGATTGGAGAAAGTTTCTAAAATATCACCTTGTGATGTTAGTTTGGAAACTTCAGATATATGTAAATGTAGTATAGGGAAGCTTCATAAGTCAGTCTCATCTGCAAATACTTGTGGGATTTTTAGCACAGCAAGTGGAAAATCTGTCCAGGTATCAGATGCTTCATTACAAAACGCAAGACAAGTGTTTTCTGAAATAGAAGATAGTACCAAGCAAGTCTTTTCCAAAGTATTGTTTAAAAGTAACGAACATTCAGACCAGCTCACAAGAGAAGAAAATACTGCTATACGTACTCCAGAACATTTAATATCCCAAAAAGGCTTTTCATATAATGTGGTAAATTCATCTGCTTTCTCTGGATTTAGTACAGCAAGTGGAAAGCAAGTTTCCATTTTAGAAAGTTCCTTACACAAAGTTAAGGGAGTGTTAGAGGAATTTGATTTAATCAGAACTGAGCATAGTCTTCACTATTCACCTACGTCTAGACAAAATGTATCAAAAATACTTCCTCGTGTTGATAAGAGAAACCCAGAGCACTGTGTAAACTCAGAAATGGAAAAAACCTGCAGTAAAGAATTTAAATTATCAAATAACTTAAATGTTGAAGGTGGTTCTTCAGAAAATAATCACTCTATTAAAGTTTCTCCATATCTCTCTCAATTTCAACAAGACAAACAACAGTTGGTATTAGGAACCAAAGTGTCACTTGTTGAGAACATTCATGTTTTGGGAAAAGAACAGGCTTCACCTAAAAACGTAAAAATGGAAATTGGTAAAACTGAAACTTTTTCTGATGTTCCTGTGAAAACAAATATAGAAGTTTGTTCTACTTACTCCAAAGATTCAGAAAACTACTTTGAAACAGAAGCAGTAGAAATTGCTAAAGCTTTTATGGAAGATGATGAACTGACAGATTCTAAACTGCCAAGTCATGCCACACATTCTCTTTTTACATGTCCCGAAAATGAGGAAATGGTTTTGTCAAATTCAAGAATTGGAAAAAGAAGAGGAGAGCCCCTTATCTTAGTGGGAGAACCCTCAATCAAAAGAAACTTATTAAATGAATTTGACAGGATAATAGAAAATCAAGAAAAATCCTTAAAGGCTTCAAAAAGCACTCCAGATGGCACAATAAAAGATCGAAGATTGTTTATGCATCATGTTTCTTTAGAGCCGATTACCTGTGTACCCTTTCGCACAACTAAGGAACGTCAAGAGATACAGAATCCAAATTTTACCGCACCTGGTCAAGAATTTCTGTCTAAATCTCATTTGTATGAACATCTGACTTTGGAAAAATCTTCAAGCAATTTAGCAGTTTCAGGACATCCATTTTATCAAGTTTCTGCTACAAGAAATGAAAAAATGAGACACTTGATTACTACAGGCAGACCAACCAAAGTCTTTGTTCCACCTTTTAAAACTAAATCACATTTTCACAGAGTTGAACAGTGTGTTAGGAATATTAACTTGGAGGAAAACAGACAAAAGCAAAACATTGATGGACATGGCTCTGATGATAGTAAAAATAAGATTAATGACAATGAGATTCATCAGTTTAACAAAAACAACTCCAATCAAGCAGTAGCTGTAACTTTCACAAAGTGTGAAGAAGAACCTTTAGATTTAATTACAAGTCTTCAGAATGCCAGAGATATACAGGATATGCGAATTAAGAAGAAACAAAGGCAACGCGTCTTTCCACAGCCAGGCAGTCTGTATCTTGCAAAAACATCCACTCTGCCTCGAATCTCTCTGAAAGCAGCAGTAGGAGGCCAAGTTCCCTCTGCGTGTTCTCATAAACAGCTGTATACGTATGGCGTTTCTAAACATTGCATAAAAATTAACAGCAAAAATGCAGAGTCTTTTCAGTTTCACACTGAAGATTATTTTGGTAAGGAAAGTTTATGGACTGGAAAAGGAATACAGTTGGCTGATGGTGGATGGCTCATACCCTCCAATGATGGAAAGGCTGGAAAAGAAGAATTTTATAGGGCTCTGTGTGACACTCCAGGTGTGGATCCAAAGCTTATTTCTAGAATTTGGGTTTATAATCACTATAGATGGATCATATGGAAACTGGCAGCTATGGAATGTGCCTTTCCTAAGGAATTTGCTAATAGATGCCTAAGCCCAGAAAGGGTGCTTCTTCAACTAAAATACAGATATGATACGGAAATTGATAGAAGCAGAAGATCGGCTATAAAAAAGATAATGGAAAGGGATGACACAGCTGCAAAAACACTTGTTCTCTGTGTTTCTGACATAATTTCATTGAGCGCAAATATATCTGAAACTTCTAGCAATAAAACTAGTAGTGCAGATACCCAAAAAGTGGCCATTATTGAACTTACAGATGGGTGGTATGCTGTTAAGGCCCAGTTAGATCCTCCCCTCTTAGCTGTCTTAAAGAATGGCAGACTGACAGTTGGTCAGAAGATTATTCTTCATGGAGCAGAACTGGTGGGCTCTCCTGATGCCTGTACACCTCTTGAAGCCCCAGAATCTCTTATGTTAAAGATTTCTGCTAACAGTACTCGGCCTGCTCGCTGGTATACCAAACTTGGATTCTTTCCTGACCCTAGACCTTTTCCTCTGCCCTTATCATCGCTTTTCAGTGATGGAGGAAATGTTGGTTGTGTTGATGTAATTATTCAAAGAGCATACCCTATACAGTGGATGGAGAAGACATCATCTGGATTATACATATTTCGCAATGAAAGAGAGGAAGAAAAGGAAGCAGCAAAATATGTGGAGGCCCAACAAAAGAGACTAGAAGCCTTATTCACTAAAATTCAGGAGGAATTTGAAGAACATGAAGAAAACACAACAAAACCATATTTACCATCACGTGCACTAACAAGACAGCAAGTTCGTGCTTTGCAAGATGGTGCAGAGCTTTATGAAGCAGTGAAGAATGCAGCAGACCCAGCTTACCTTGAGGGTTATTTCAGTGAAGAGCAGTTAAGAGCCTTGAATAATCACAGGCAAATGTTGAATGATAAGAAACAAGCTCAGATCCAGTTGGAAATTAGGAAGGCCATGGAATCTGCTGAACAAAAGGAACAAGGTTTATCAAGGGATGTCACAACCGTGTGGAAGTTGCGTATTGTAAGCTATTCAAAAAAAGAAAAAGATTCAGTTATACTGAGTATTTGGCGTCCATCATCAGATTTATATTCTCTGTTAACAGAAGGAAAGAGATACAGAATTTATCATCTTGCAACTTCAAAATCTAAAAGTAAATCTGAAAGAGCTAACATACAGTTAGCAGCGACAAAAAAAACTCAGTATCAACAACTACCGGTTTCAGATGAAATTTTATTTCAGATTTACCAGCCACGGGAGCCCCTTCACTTCAGCAAATTTTTAGATCCAGACTTTCAGCCATCTTGTTCTGAGGTGGACCTAATAGGATTTGTCGTTTCTGTTGTGAAAAAAACAGGACTTGCCCCTTTCGTCTATTTGTCAGACGAATGTTACAATTTACTGGCAATAAAGTTTTGGATAGACCTTAATGAGGACATTATTAAGCCTCATATGTTAATTGCTGCAAGCAACCTCCAGTGGCGACCAGAATCCAAATCAGGCCTTCTTACTTTATTTGCTGGAGATTTTTCTGTGTTTTCTGCTAGTCCAAAAGAGGGCCACTTTCAAGAGACATTCAACAAAATGAAAAATACTGTTGAGAATATTGACATACTTTGCAATGAAGCAGAAAACAAGCTTATGCATATACTGCATGCAAATGATCCCAAGTGGTCCACCCCAACTAAAGACTGTACTTCAGGGCCGTACACTGCTCAAATCATTCCTGGTACAGGAAACAAGCTTCTGATGTCTTCTCCTAATTGTGAGATATATTATCAAAGTCCTTTATCACTTTGTATGGCCAAAAGGAAGTCTGTTTCCACACCTGTCTCAGCCCAGATGACTTCAAAGTCTTGTAAAGGGGAGAAAGAGATTGATGACCAAAAGAACTGCAAAAAGAGAAGAGCCTTGGATTTCTTGAGTAGACTGCCTTTACCTCCACCTGTTAGTCCCATTTGTACATTTGTTTCTCCGGCTGCACAGAAGGCATTTCAGCCACCAAGGAGTTGTGGCACCAAATACGAAACACCCATAAAGAAAAAAGAACTGAATTCTCCTCAGATGACTCCATTTAAAAAATTCAATGAAATTTCTCTTTTGGAAAGTAATTCAATAGCTGACGAAGAACTTGCATTGATAAATACCCAAGCTCTTTTGTCTGGTTCAACAGGAGAAAAACAATTTATATCTGTCAGTGAATCCACTAGGACTGCTCCCACCAGTTCAGAAGATTATCTCAGACTGAAACGACGTTGTACTACATCTCTGATCAAAGAACAGGAGAGTTCCCAGGCCAGTACGGAAGAATGTGAGAAAAATAAGCAGGACACAATTACAACTAAAAAATATATCTAA

**>BRCA2 WT protein (3418AA)**

MPIGSKERPTFFEIFKTRCNKADLGPISLNWFEELSSEAPPYNSEPAEESEHKNNNYEPNLFKTPQRKPSYNQLASTPIIFKEQGLTLPLYQSPVKELDKFKLDLGRNVPNSRHKSLRTVKTKMDQADDVSCPLLNSCLSES**P**VVLQCTHVTPQRDKSVVCGSLFHTPKFVKGRQTPKHISESLGAEVDPDMSWSSSLATPPTLSSTVLIVRNEEASETVFPHDTTANVKSYFSNHDESLKKNDRFIASVTDSENTNQREAASHGFGKTSGNSFKVNSCKDHIGKSMPNVLEDEVYETVVDTSEEDSFSLCFSKCRTKNLQKVRTSKTRKKIFHEANADECEKSKNQVKEKYSFVSEVEPNDTDPLDSNVANQKPFESGSDKISKEVVPSLACEWSQLTLSGLNGAQMEKIPLLHISSCDQNISEKDLLDTENKRKKDFLTSENSLPRISSLPKSEKPLNEETVVNKRDEEQHLESHTDCILAVKQAISGTSPVASSFQGIKKSIFRIRESPKETFNASFSGHMTDPNFKKETEASESGLEIHTVCSQKEDSLCPNLIDNGSWPATTTQNSVALKNAGLISTLKKKTNKFIYAIHDETSYKGKKIPKDQKSELINCSAQFEANAFEAPLTFANADSGLLHSSVKRSCSQNDSEEPTLSLTSSFGTILRKCSRNETCSNNTVISQDLDYKEAKCNKEKLQLFITPEADSLSCLQEGQCENDPKSKKVSDIKEEVLAAACHPVQHSKVEYSDTDFQSQKSLLYDHENASTLILTPTSKDVLSNLVMISRGKESYKMSDKLKGNNYESDVELTKNIPMEKNQDVCALNENYKNVELLPPEKYMRVASPSRKVQFNQNTNLRVIQKNQEETTSISKITVNPDSEELFSDNENNFVFQVANERNNLALGNTKELHETDLTCVNEPIFKNSTMVLYGDTGDKQATQVSIKKDLVYVLAEENKNSVKQHIKMTLGQDLKSDISLNIDKIPEKNNDYMNKWAGLLGPISNHSFGGSFRTASNKEIKLSEHNIKKSKMFFKDIEEQYPTSLACVEIVNTLALDNQKKLSKPQSINTVSAHLQSSVVVSDCKNSHITPQMLFSKQDFNSNHNLTPSQKAEITELSTILEESGSQFEFTQFRKPSYILQKSTFEVPENQMTILKTTSEECRDADLHVIMNAPSIGQVDSSKQFEGTVEIKRKFAGLLKNDCNKSASGYLTDENEVGFRGFYSAHGTKLNVSTEALQKAVKLFSDIENISEETSAEVHPISLSSSKCHDSVVSMFKIENHNDKTVSEKNNKCQLILQNNIEMTTGTFVEEITENYKRNTENEDNKYTAASRNSHNLEFDGSDSSKNDTVCIHKDETDLLFTDQHNICLKLSGQFMKEGNTQIKEDLSDLTFLEVAKAQEACHGNTSNKEQLTATKTEQNIKDFETSDTFFQTASGKNISVAKESFNKIVNFFDQKPEELHNFSLNSELHSDIRKNKMDILSYEETDIVKHKILKESVPVGTGNQLVTFQGQPERDEKIKEPTLLGFHTASGKKVKIAKESLDKVKNLFDEKEQGTSEITSFSHQWAKTLKYREACKDLELACETIEITAAPKCKEMQNSLNNDKNLVSIETVVPPKLLSDNLCRQTENLKTSKSIFLKVKVHENVEKETAKSPATCYTNQSPYSVIENSALAFYTSCSRKTSVSQTSLLEAKKWLREGIFDGQPERINTADYVGNYLYENNSNSTIAENDKNHLSEKQDTYLSNSSMSNSYSYHSDEVYNDSGYLSKNKLDSGIEPVLKNVEDQKNTSFSKVISNVKDANAYPQTVNEDICVEELVTSSSPCKNKNAAIKLSISNSNNFEVGPPAFRIASGKIVCVSHETIKKVKDIFTDSFSKVIKENNENKSKICQTKIMAGCYEALDDSEDILHNSLDNDECSTHSHKVFADIQSEEILQHNQNMSGLEKVSKISPCDVSLETSDICKCSIGKLHKSVSSANTCGIFSTASGKSVQVSDASLQNARQVFSEIEDSTKQVFSKVLFKSNEHSDQLTREENTAIRTPEHLISQKGFSYNVVNSSAFSGFSTASGKQVSILESSLHKVKGVLEEFDLIRTEHSLHYSPTSRQNVSKILPRVDKRNPEHCVNSEMEKTCSKEFKLSNNLNVEGGSSENNHSIKVSPYLSQFQQDKQQLVLGTKVSLVENIHVLGKEQASPKNVKMEIGKTETFSDVPVKTNIEVCSTYSKDSENYFETEAVEIAKAFMEDDELTDSKLPSHATHSLFTCPENEEMVLSNSRIGKRRGEPLILVGEPSIKRNLLNEFDRIIENQEKSLKASKSTPDGTIKDRRLFMHHVSLEPITCVPFRTTKERQEIQNPNFTAPGQEFLSKSHLYEHLTLEKSSSNLAVSGHPFYQVSATRNEKMRHLITTGRPTKVFVPPFKTKSHFHRVEQCVRNINLEENRQKQNIDGHGSDDSKNKINDNEIHQFNKNNSNQAVAVTFTKCEEEPLDLITSLQNARDIQDMRIKKKQRQRVFPQPGSLYLAKTSTLPRISLKAAVGGQVPSACSHKQLYTYGVSKHCIKINSKNAESFQFHTEDYFGKESLWTGKGIQLADGGWLIPSNDGKAGKEEFYRALCDTPGVDPKLISRIWVYNHYRWIIWKLAAMECAFPKEFANRCLSPERVLLQLKYRYDTEIDRSRRSAIKKIMERDDTAAKTLVLCVSDIISLSANISETSSNKTSSADTQKVAIIELTDGWYAVKAQLDPPLLAVLKNGRLTVGQKIILHGAELVGSPDACTPLEAPESLMLKISANSTRPARWYTKLGFFPDPRPFPLPLSSLFSDGGNVGCVDVIIQRAYPIQWMEKTSSGLYIFRNEREEEKEAAKYVEAQQKRLEALFTKIQEEFEEHEENTTKPYLPSRALTRQQVRALQDGAELYEAVKNAADPAYLEGYFSEEQLRALNNHRQMLNDKKQAQIQLEIRKAMESAEQKEQGLSRDVTTVWKLRIVSYSKKEKDSVILSIWRPSSDLYSLLTEGKRYRIYHLATSKSKSKSERANIQLAATKKTQYQQLPVSDEILFQIYQPREPLHFSKFLDPDFQPSCSEVDLIGFVVSVVKKTGLAPFVYLSDECYNLLAIKFWIDLNEDIIKPHMLIAASNLQWRPESKSGLLTLFAGDFSVFSASPKEGHFQETFNKMKNTVENIDILCNEAENKLMHILHANDPKWSTPTKDCTSGPYTAQIIPGTGNKLLMSSPNCEIYYQSPLSLCMAKRKSVSTPVSAQMTSKSCKGEKEIDDQKNCKKRRALDFLSRLPLPPPVSPICTFVSPAAQKAFQPPRSCGTKYETPIKKKELNSPQMTPFKKFNEISLLESNSIADEELALINTQALLSGSTGEKQFISVSESTRTAPTSSEDYLRLKRRCTTSLIKEQESSQASTEECEKNKQDTITTKKYI

**>BRCA2 c.475+5G>C protein (3418AA)**

MPIGSKERPTFFEIFKTRCNKADLGPISLNWFEELSSEAPPYNSEPAEESEHKNNNYEPNLFKTPQRKPSYNQLASTPIIFKEQGLTLPLYQSPVKELDKFKLDLGRNVPNSRHKSLRTVKTKMDQADDVSCPLLNSCLSES**G**MWEFVSYTKVCEGSSDTKTYF-KSRS-GGS-YVLVKFFSYTTHP-FYCAHSQK-RSI-NCISS-YYC-CEKLFFQS--KSEEK--IYRFCDRQ-KHKSKRSCKSWIWKNIREFI-SK-LQRPHWKVNAKCPRR-SI-NSCRYL-RR-FFIMFF-M-NKKSTKSKN-QD-EKNFP-SKR--M-KI-KPSERKILICI-SGTK-Y-SIRFKCSKSEAL-EWK-QNLQGSCTVFGL-MVSTNPFRSKWSPDGENTPIAYFFM-PKYFRKRPIRHREQKKERFSYFREFFATYF-PTKIREAIK-GNSGK-ER-RAAS-ISYRLHSCSKAGNIWNFSSGFFISGYQKVYIQNKRIT-RDFQCKFFRSYD-SKL-KRN-SL-KWTGNTYCLLTEGGLLMSKFN--WKLASHHHTEFCSFEECRFNIHFEKENK-VYLCYT--NIL-RKKNTERPKIRTN-LFSPV-SKCF-STTYICKC-FRFIAFFCEKKLFTE-F-RTNFVLN-LFWDNSEEMF-K-NMF--YSNLSGS-L-RSKM--GKTTVIYYPRS-FSVMPAGRTV-K-SKKQKSFRYKRRGLGCSMSPSTTFKSGIQ-Y-LSIPEKSFI-S-KCQHSYFNSYFQGCSVKPSHDF-RQRIIQNVRQAQR-QL-I-C-INQKYSHGKESRCMCFK-KL-KR-AVAT-KIHESSITFKKGTIQPKHKSKSNPKKSRRNYFNFKNNCQSRL-RTFLRQ-E-FCLPSS--KE-SCFRKY-GTS-NRLDLCKRTHFQELYHGFIWRHR--TSNPSVN-KRFGLCSCRGEQK-CKAAYKNDSRSRFKIGHLLEYR-NTRKK--LHEQMGRTLRSNFKSQFWR-LQNSFK-GNQAL-T-H-EEQNVLQRY-RTISY-FSLC-NCKYLGIR-SKETEQASVN-YCICTFTE-CSCF-L-K-SYNPSDVIFQAGF-FKP-FNT-PKGRNYRTFYYIRRIRKSV-IYSV-KTKLHIAEEYI-SA-KPDDYLKDHF-GMQRC-SSCHNECPIDWSGRQQQAI-RYS-N-TEVCWPVEK-L-QKCFWLFNR-K-SGV-GLLFCSWHKTECFY-SSAKSCETV--Y-EY--GNFCRGTSNKFIFK-MS-FCCFNV-DRKS---NCK-KK--MPTDITK-Y-NDYWHFC-RNY-KLQEKY-K-R-QIYCCQ-KFS-LRI-WQ-FK-K-YCLYS-R-NGLAIY-SAQHMS-IIWPVYEGGKHSD-RRFVRFNFFGSCESSRSMSW-YFK-RTVNCY-NGAKYKRF-DF-YIFSDCKWEKY-CRQRVI--NCKFL-SETRRIA-LFLKF-ITF-HKKEQNGHSKL-GNRHS-TQNTERKCPSWYWKSTSDLPGTTRT--KDQRTYSIGFSYS-REKS-NCKGIFGQSEKPF--KRARY--NHQF-PSMGKDPKVQRGL-RP-ISM-DH-DHSCPKV-RNAEFSQ---KPCFY-DCGAT-ALK--FM-TN-KSQNIKKYLFES-ST-KCRKRNSKKSCNLLHKSVPLFSH-KFSLSFLHKL--KNFCESDFIT-SKKMA-RRNI-WSTRKNKYCRLCRKLFV-K-FKQYYS-K-QKSSLRKTRYLFK-Q-HV-QLFLPF--GI--FRISLKK-T-FWY-ASIEEC-RSKKH-FFQSNIQCKRCKCIPTNCK-RYLR-GTCD-LFTLQK-KCSH-IVHI----F-GRATCI-DSQW-NRLCFT-NN-KSERHIYRQFQ-SN-GKQRE-IKNLPNENYGRLLRGIG-FRGYSS-LSR---M-HAFT-GFC-HSE-RNFTT-PKYVWIGESF-NITL-C-FGNFRYM-M-YREAS-VSLICKYLWDF-HSKWKICPGIRCFITKRKTSVF-NRR-YQASLFQSIV-K-RTFRPAHKRRKYCYTYSRTFNIPKRLFI-CGKFICFLWI-YSKWKASFHFRKFLTQS-GSVRGI-FNQN-A-SSLFTYV-TKCIKNTSSC--EKPRALCKLRNGKNLQ-RI-IIK-LKC-RWFFRK-SLY-SFSISLSISTRQTTVGIRNQSVTC-EHSCFGKRTGFT-KRKNGNW-N-NFF-CSCENKYRSLFYLLQRFRKLL-NRSSRNC-SFYGR--TDRF-TAKSCHTFSFYMSRK-GNGFVKFKNWKKKRRAPYLSGRTLNQKKLIK-I-QDNRKSRKILKGFKKHSRWHNKRSKIVYASCFFRADYLCTLSHN-GTSRDTESKFYRTWSRISV-ISFV-TSDFGKIFKQFSSFRTSILSSFCYKK-KNETLDYYRQTNQSLCSTF-N-ITFSQS-TVC-EY-LGGKQTKAKH-WTWL----K-D--Q-DSSV-QKQLQSSSSCNFHKV-RRTFRFNYKSSECQRYTGYAN-EETKATRLSTARQSVSCKNIHSASNLSESSSRRPSSLCVFS-TAVYVWRF-TLHKN-QQKCRVFSVSH-RLFW-GKFMDWKRNTVG-WWMAHTLQ-WKGWKRRIL-GSV-HSRCGSKAYF-NLGL-SL-MDHMETGSYGMCLS-GIC--MPKPRKGASSTKIQI-YGN--KQKIGYKKDNGKG-HSCKNTCSLCF-HNFIERKYI-NF-Q-N--CRYPKSGHY-TYRWVVCC-GPVRSSPLSCLKEWQTDSWSEDYSSWSRTGGLS-CLYTS-SPRISYVKDFC-QYSACSLVYQTWILS-P-TFSSALIIAFQ-WRKCWLC-CNYSKSIPYTVDGEDIIWIIHISQ-KRGRKGSSKICGGPTKETRSLIH-NSGGI-RT-RKHNKTIFTITCTNKTASSCFARWCRAL-SSEECSRPSLP-GLFQ-RAVKSLE-SQANVE--ETSSDPVGN-EGHGIC-TKGTRFIKGCHNRVEVAYCKLFKKRKRFSYTEYLASIIRFIFSVNRRKEIQNLSSCNFKI-K-I-KS-HTVSSDKKNSVSTTTGFR-NFISDLPATGAPSLQQIFRSRLSAILF-GGPNRICRFCCEKNRTCPFRLFVRRMLQFTGNKVLDRP--GHY-ASYVNCCKQPPVATRIQIRPSYFICWRFFCVFC-SKRGPLSRDIQQNEKYC-EY-HTLQ-SRKQAYAYTACK-SQVVHPN-RLYFRAVHCSNHSWYRKQASDVFS-L-DILSKSFITLYGQKEVCFHTCLSPDDFKVL-RGERD--PKELQKEKSLGFLE-TAFTSTC-SHLYICFSGCTEGISATKELWHQIRNTHKEKRTEFSSDDSI-KIQ-NFSFGK-FNS-RRTCIDKYPSSFVWFNRRKTIYICQ-IH-DCSHQFRRLSQTETTLYYISDQRTGEFPGQYGRM-EK-AGHNYN-KIYL

**p. (Pro143Glyfs)**
